# Two Types of Temporal Symmetry in the Laws of Nature

**DOI:** 10.3390/e27050466

**Published:** 2025-04-25

**Authors:** A. Y. Klimenko

**Affiliations:** Centre for Multiscale Energy Systems, School of Mechanical and Mining Engineering, The University of Queensland, St. Lucia, Brisbane 4072, Australia; a.klimenko@uq.edu.au

**Keywords:** temporal symmetry, Markov diffusion bridge, arrow of time, entropy increase

## Abstract

This work explores the implications of assuming time symmetry and applying bridge-type, time-symmetric temporal boundary conditions to deterministic laws of nature with random components. The analysis, drawing on the works of Kolmogorov and Anderson, leads to two forms of governing equations, referred to here as symmetric and antisymmetric. These equations account for the emergence of characteristics associated with conventional thermodynamics, the arrow of time, and a form of antecedent causality. The directional properties of time arise from the mathematical structure of Markov bridges in proximity of the corresponding temporal boundary conditions, without requiring any postulates that impose a preferred direction of time.

## 1. Introduction

The *arrow of time* remains one of the most evident and most guarded secrets of nature. In this context, major theories and models can be divided into two groups: (1) *time-directional*, which recognises inequivalence of the directions of time, and (2) *time-symmetric*, which implies that the directions of time are conceptually equivalent. Thermodynamics, chemical kinetics, and viscous and diffusive fluid mechanics belong to the first group, which explicitly incorporate time-directional, irreversible evolutions. For example, the second law of thermodynamics states that the entropy of an isolated system must not decrease as time moves forward. In contrast, classical mechanics, relativistic mechanics, unitary quantum mechanics, and electromagnetism are fundamentally time-reversible. In these theories, the directionality of time is conventionally introduced through *antecedent causality*. While causality reflects a general scientific principle of uncovering deep underlying connections between events or processes, antecedent causality specifically emphasises the temporal precedence of causes over effects. Although there are various interpretations of causality [1,2,3,4], our understanding of antecedent causality is focused on the conceptual and practical preference for specifying *initial* rather than *final* conditions. This preference discriminates the directions of time (i.e., introduces the *arrow of time*) directly or with common corollaries, such as that dependencies appear after but not before interactions or that an intervention is followed by—but not preceded by—its effect. The combination of time-symmetric theories with antecedent causality often allows the demonstration of entropy increase, as is performed in Boltzmann’s H-theorem [5] or Zurek’s decoherence theory [6].

While antecedent causality is deeply embedded in our intuition and remains implicitly present in most theories and models, beginning with Hume [1], Boltzmann [5], and Russell [2], it is commonly accepted that antecedent causality is a valuable intuitive tool but not a necessity of thought or the most fundamental underlying property of nature. Starting with Boltzmann [5] and Reichenbach [3], many philosophers tend to define or relate antecedent causality to the time-directional properties of the second law. This leads to the fundamental *logical circuit*: antecedent causality is defined in relation to the entropy increase and entropy increase is demonstrated using antecedent causality [7].

If, for analysing the direction of time, we wish to avoid a priori discrimination of the directions of time (which is rather difficult due to our well-developed temporal intuition [8]), the practice of explicitly or implicitly imposing initial (and not final) conditions must give way to *time-symmetric temporal boundary conditions* jointly set in the past and in the future. The two-state vector formalism in quantum mechanics [9] is a good example of a model that offers such time-symmetric interpretation of laws of nature by representing the current state as a combination of two quantum waves: $|\varphi \rangle$ propagating forward in time and satisfying some initial conditions ${|\varphi \rangle }_{t=t_{1}}=\varphi_{1}$, and $\langle \psi |$ propagating backward in time and satisfying some final conditions ${\langle \psi |}_{t=t_{2}}=\psi_{2}$, where $t_{2}>t_{1}.$ The product 〈ψ|φ〉 is then proportional to a probability, which somewhat resembles the structure of the probability equations presented in this work. It should be noted that, unlike the present approach, the two-state vector formalism—being based on unitary transformations—cannot account for irreversible effects or generate an arrow of time. Alternatively, Drummond [10] showed that the unitary evolution of a quantum field can be reformulated as a time-symmetric Fokker–Planck equation, allowing for both forward and backward stochastic propagation. While Drummond’s approach bears some mathematical resemblance to our present treatment, the physical role of randomness that concerns us—priming thermodynamic irreversibilities—is entirely different.

The relationship between the thermodynamic arrow of time and cosmic dynamics was promoted by Gold [11], who suggested that the expansion of the universe is associated with increasing entropy and, therefore, that contraction should correspond to decreasing entropy. Hawking followed this line of reasoning, proposing similar gravitational treatments of the Big Bang and the Big Crunch. However, their approach was criticised by Penrose, who argued that even if the universe were to undergo a Big Crunch, it would have high entropy, making it fundamentally different from the Big Bang [12]. Schulman [13] explained that it is not the expansion or contraction of the universe that determines the arrow of time, but rather the temporal boundary conditions. While it is common to assume that the initial state of the universe—the Big Bang—had low entropy, Schulman proposed that the final state—the Big Crunch—may also have low entropy. Although Gold’s and Schulman’s approaches may exhibit similar global entropy dynamics, they rest on different physical foundations and carry distinct implications. Tamm [14] considered cosmological consequences of having both a low-entropy Big Bang and a low-entropy Big Crunch, although our focus here is on the thermodynamic rather than the cosmological aspects.

The aim of this work is to examine the implications of Schulman’s formulation [13,15], which imposes two temporal boundary conditions. The present model shares a number of features and some implications with Schulman’s approach. However, unlike Schulman’s original framework, we assume the intrinsic presence of *randomness* in nature. This implies that even the smallest thermodynamic systems are ubiquitously affected by randomness, which is considered to be ultimately responsible for the arrow of time and that entropy is an objective, observer-independent quantity. This assumption resolves the technical difficulties associated with simultaneously imposing two boundary conditions in deterministic systems (which requires dynamical mixing on galactic scales), and naturally leads to *stochastic bridge* formulations. The term *bridge* conventionally refers to stochastic processes constrained at both the initial and final times, while *stochastic* denotes genuine randomness rather than deterministic chaos. Our goal is to construct a time-symmetric stochastic model subject to time-symmetric boundary conditions, which is nonetheless capable of explaining irreversible behaviour in nature and, effectively, producing the arrow of time reflecting the influence of the nearest temporal boundary conditions. This model avoids postulating antecedent causality and instead points to physical mechanisms that can give rise to irreversible effects and exhibit features conventionally associated with causal behaviour.

Section 2 considers the laws of Hamiltonian mechanics (classical and quantum) and introduces two types of temporal symmetry: odd and even. Section 3 addresses random models under bridge conditions. Section 4 and Section 5 explore the physical implications of these models. Conclusions are presented in Section 6. The Appendix A and Appendix B cover the mathematical properties of Markov diffusion bridges and examines temporal reversibility of stochastic processes.

## 2. The Laws of Mechanics and Determinism

An increase in entropy corresponds to a loss of information and an increase in uncertainty, which implies randomness. If one believes that the thermodynamic arrow of time is real, so too must randomness be real, which from a philosophical perspective is assumed to be *ontological* rather than *epistemic*. Randomness can be interpreted in two main ways: ontological (real, true) randomness, where random events occur inherently and are not entirely determined by prior deterministic causes, and epistemic or subjective randomness, where the world remains deterministic while unpredictability arises from a lack of knowledge about the system. This work adopts the ontological interpretation of randomness, asserting that it is not merely a reflection of incomplete knowledge, but a fundamental aspect of nature—inherently present everywhere and responsible for time-directional effects. In this view, the arrow of time and entropy increase are not just apparent properties but are intrinsically linked to the irreducible stochastic character of the laws of nature. The ontological perspective views entropy as a real physical quantity, rather than something that depends on subjective interpretations (such as coarse-graining). Yet, we also recognise the importance of deterministic laws in establishing fundamental constraints and governing principles that shape the evolution of physical systems, restrain randomness, and impose symmetries.

Consider a system of a very high dimension

(1) $$\frac{dx^{i}}{dt}=a^{i}\left(x,t\right)+b^{ik}\left(x,t\right)\xi^{k}\left(t\right)$$

reflecting the known laws of nature, where $x^{i}$ can represent generalised coordinates, momenta, or any other dynamic parameters as needed. We assume that these laws are discrete (e.g., represented by dynamics of discrete particles) or allow for discretisation. The Einstein summation convention applies here and in the rest of the paper. The first term, $a^{i}\left(x,t\right)$ reflects deterministic mechanical laws (including classical, relativistic, and quantum mechanics), which are considered time-reversible and, on their own, do not possess a time arrow. The second term involves a genuinely random quantity (specifically, white noise), which is responsible for time-directional effects. This term is generally assumed to be very small, such that deterministic laws typically exhibit high precision; yet, this small random component serves as a *time primer*—the process ultimately responsible for the emergence of irreversibility. Dynamical mixing in complex mechanical or quantum systems can amplify the thermodynamic consequences of these random contributions. Nevertheless, the realistic nature of randomness underpins the presence of such subtle effects in every minute system we may wish to consider.

Let us put $b^{ik}=0$ and examine common properties of the deterministic laws.

### 2.1. Mechanical Laws

The essence of mechanical laws is conventionally expressed by canonical *Hamiltonian equations*. Assume x={q,p}, then $a=\{\dot{q},\dot{p}\}$ and canonical equations become [16]

(2) $${\dot{q}}^{i}\overset{\mathrm{def}}{=}\frac{dq^{i}}{dt}=\frac{\partial H}{\partial p_{i}}=a_{\left(q\right)}^{i},\;\;\;{\dot{p}}_{i}\overset{\mathrm{def}}{=}\frac{dp_{i}}{dt}=-\frac{\partial H}{\partial q^{i}}=a_{i}^{\left(p\right)}$$

implying

(3) $$\frac{\partial {\dot{q}}^{i}}{\partial q^{i}}+\frac{\partial {\dot{p}}_{i}}{\partial p_{i}}=0$$

leading to $\partial a^{i}/\partial x^{i}=0$. This condition is important to ensure that the deterministic equations do not produce entropy and/or lose information and, therefore, can always be reversed and solved backward in time. Hamiltonian equations possess some time-symmetric properties: substituting $\bar{t}=-t,$ ${\bar{q}}^{i}=q^{i}$ and ${\bar{p}}^{i}=-p^{i}$ into (2) yields the system

(4) $$\frac{d{\bar{q}}^{i}}{d\bar{t}}=\frac{\partial H}{\partial {\bar{p}}_{i}},\;\;\;\frac{d{\bar{p}}^{i}}{d\bar{t}}=-\frac{\partial H}{\partial {\bar{q}}^{i}}$$

which remains invariant, provided *H* depends on $p^{2}$ and not on p. While this is true in many cases, some systems (e.g., involving rotating frames or magnetic field) have terms that are linear with respect to $p^{i}$. In this case, invariant time reversal requires reversal of rotation and inverse of the direction of magnetic field (or charge conjugation).

In *relativistic mechanics*, *t* is replaced by proper time τ while the structure of the Hamiltonian equations remains the same. The definition of the proper time in relativity $d\tau^{2}=g_{\nu \mu }dx^{\nu }dx^{\mu }$ in terms of four-vectors $dx^{\nu }$ and the metric tensor $g_{\nu \mu }$ has an arbitrary sign ${\left(-d\tau \right)}^{2}={\left(+d\tau \right)}^{2}$ and, therefore, relativistic equations tend to be fully invariant with respect to the proper time reversal τ−>−τ [16].

### 2.2. Laws of Quantum Mechanics

In quantum mechanics, the *Hamiltonian form* of the governing equation iℏ(∂Ψ/∂t)=HΨ is complex and needs to be separated into real and imaginary parts to match our models [16]

(5) $$\left\{\begin{array}{c} {\dot{q}}^{i}\overset{\mathrm{def}}{=}\frac{\partial q^{i}}{\partial t}=\frac{1}{\hbar }\left(H_{s}^{ij}p^{j}+H_{a}^{ij}q^{j}\right)=a_{q}^{i}\\ {\dot{p}}^{i}\overset{\mathrm{def}}{=}\frac{\partial p^{i}}{\partial t}=\frac{-1}{\hbar }\left(H_{s}^{ij}q^{j}-H_{a}^{ij}p^{j}\right)=a_{p}^{i} \end{array}\right.$$

where $\Psi =\Sigma_{j}\left(q^{j}+ip^{j}\right)|j\rangle$ and $H_{s}$ reflects symmetric and $H_{a}$ reflects antisymmetric components of the Hamiltonian operator so that $H=H_{s}+iH_{a}$ is Hermitian and evolution specified by (5) is unitary. Evaluation of divergence yields

(6) $$\frac{\partial {\dot{q}}^{i}}{\partial q^{i}}+\frac{\partial {\dot{p}}^{i}}{\partial p^{i}}=H_{a}^{ii}+H_{a}^{ii}=2\mathrm{trace}\left(H_{a}\right)=0$$

again leading to $\partial a^{i}/\partial x^{i}=0$. If the basis is selected so that the antiunitary time reversal operator T=KU is represented in this basis by complex conjugation K, then temporal symmetry requires that the Hamiltonian is real $H=H_{s}$ and symmetric, while $H_{a}=0$. The time reversal of (5) yields

(7) $$\left\{\begin{array}{c} \frac{\partial {\bar{q}}^{i}}{\partial \bar{t}}=\frac{1}{\hbar }\left(H_{s}^{ij}{\bar{p}}^{j}\right)\\ \frac{\partial {\bar{p}}^{i}}{\partial \bar{t}}=\frac{-1}{\hbar }\left(H_{s}^{ij}{\bar{q}}^{j}\right) \end{array}\right.$$

where $\bar{t}=-t,$ ${\bar{q}}^{i}=q^{i}$ and ${\bar{p}}^{i}=-p^{i}$.

While non-relativistic quantum mechanics considers time reversal as an external transformation, relativistic quantum mechanics tends to possess direct-time and reverse-time solutions, with the former associated with particles and the latter with antiparticles. The modern view in quantum field theory is that the fundamental laws of the universe are not completely time-symmetric, although they are close to being time-symmetric. Specifically, the governing equations remain exactly the same under CPT transformation, i.e., when time reversal is combined with parity transformation (spatial inversion) and charge conjugation (swapping matter with antimatter).

### 2.3. Time Reversal in Deterministic Equations

Overall, the basic deterministic physical laws governing the universe tend to preserve phase space volume (are non-divergent)

(8) $$\frac{\partial a^{i}}{\partial x^{i}}=0$$

making evolutions governed by these laws physically time reversible

(9) $$\left(A1\right)\;\frac{dx^{i}}{dt}=a^{i}\left(x,t\right)\Leftrightarrow \left(A2\right)\frac{dx^{i}}{d\bar{t}}={\bar{a}}^{i}\left(x,t\right)=-a^{i}\left(x,t\right),\;\;\;\bar{t}=-t\;$$

While this equation is a mathematical identity, equivalence of A1 and A2 also has a subtle physical implication: information should not be not lost and entropy should be preserved by these equations so that they can be equivalently solved forward in time or backward in time. Time reversal A is reversal of the order of the events without any other changes.

We also consider another type of time reversal, requiring that the governing laws remain the same when time is reversed, implying

(10) $$\left(B1\right)\;\frac{dx^{i}}{dt}=a^{i}\left(x\right)\Leftrightarrow \left(B2\right)\frac{d{\bar{x}}^{i}}{d\bar{t}}={\bar{a}}^{i}\left(\bar{x}\right),\;\;{\bar{a}}^{i}\left(x\right)=a^{i}\left(x\right),\;\;\;\bar{t}=-t$$

In this case, the parameters of the model should not explicitly depend on time since this would immediately create asymmetry of the directions of time. Here, ${\bar{x}}^{i}\neq x^{i}$ generally, but there should be a simple transformation $\bar{x}=Fx$ (or $x=\bar{F}\bar{x},$ $\bar{F}F=I$), which involves flipping signs and, possibly, some permutations. Replacing variables in B1 results in $d\bar{x}/d\bar{t}=$ −Fa(x), since a transforms contravariantly. Substituting $x=\bar{F}\bar{x}$ yields $-Fa\left(\bar{F}\bar{x}\right)=\bar{a}\left(\bar{x}\right)=a\left(\bar{x}\right)$ to be consistent with B2. Finally, we obtain conditions $a\left(\bar{F}\bar{x}\right)=-\bar{F}a\left(\bar{x}\right)$ and $a\left(Fx\right)=-Fa\left(x\right)$ for consistency between B1 and B2. It is this relation that constitutes the temporal symmetry: without F-consistency, B1 and B2 would simply represent different models. Examples are given in our previous analysis (2): x={q,p}, and $\bar{x}=\left\{\bar{q},\bar{p}\right\}$, where $\bar{q}=q$ and $\bar{p}=-p$ defines F. The mechanical laws generally imply existence of B-type time symmetry but require adjustments by F and may need further qualifications. While B1 and B2 may represent similar but physically different systems, A1 and A2 can usually be seen as evolutions of the same system examined from different perspectives. The B-type symmetry can often be approximate and swapping some variables might be needed to make it exact or more accurate. For example, B-type CPT-invariant reversal of time in the universe would require all its matter replaced by antimatter (i.e., swapping the corresponding matter and antimatter states)—this is only conceptual but not physical possibility.

While in dynamic equations A-type transformation is reversal of time and B-type is more related to temporal symmetries, the terms *reversal*, *reversibility*, and *temporal symmetry* are commonly used interchangeably: in stochastic systems, the A-type reversal implies presence of some symmetry, while B-type symmetry involves (or can involve) time reversal and is referred to as reversibility in a number of principal works. As we need to clearly distinguish these cases, the B-type time symmetry is referred to as *even* and the associated transformations are called *symmetric*, while A-type symmetry is referred to as *odd* and the associated transformations as *antisymmetric*. The term *temporal symmetry* and the adjective *time-symmetric* (used in the Introduction) imply general presence of symmetry without referring to a specific type. As can be seen in the rest of the paper, both types involve reversal of time and possess some distinct temporal symmetries—yet, they produce different models. Since these models are connected to both symmetry and transformations, even and symmetric on one side, and odd and antisymmetric on the other, can be used interchangeably.

**Figure 1:**
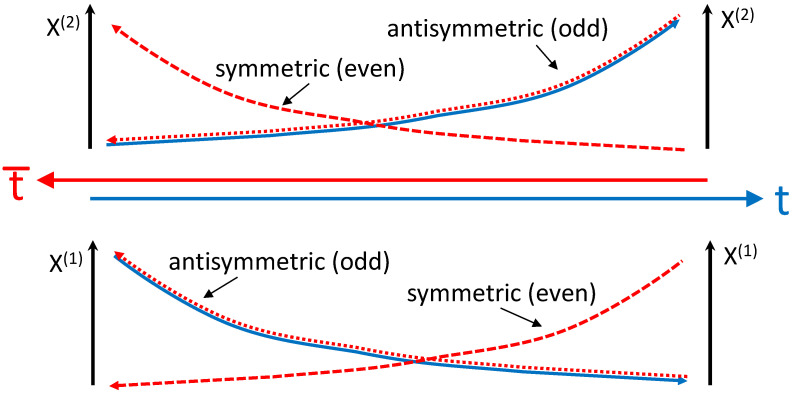
Illustration of symmetric and antisymmetric time reversal. Forward-time process (solid), symmetric reverse (dashed), antisymmetric reverse (dotted).

The following two-dimensional example, which is schematically presented in Figure 1, illustrates the difference between symmetric and antisymmetric reversal of time. Assume $b^{i}=0$ and $a^{\left(1\right)}=-x^{\left(1\right)},$ $a^{\left(2\right)}=+x^{\left(2\right)}$ corresponding to a converging-diverging velocity field complying with (8) and specifying trajectories $x^{\left(1\right)}∼e^{-t}$ and $x^{\left(2\right)}∼e^{+t}$. The antisymmetric time reversal ${\bar{a}}^{\left(1\right)}=+x^{\left(1\right)},$ ${\bar{a}}^{\left(2\right)}=-x^{\left(2\right)}$ reproduces the same trajectories $x^{\left(1\right)}∼e^{+\bar{t}}=e^{-t}$ and $x^{\left(2\right)}∼e^{-\bar{t}}=e^{+t},$ so that converging becomes diverging backward in time and vice versa. The symmetric time reverse ${\bar{a}}^{\left(1\right)}=-x^{\left(1\right)},$ ${\bar{a}}^{\left(2\right)}=+x^{\left(2\right)}$ produces evenly similar trajectories in reversed time $x^{\left(1\right)}∼e^{-\bar{t}}$ and $x^{\left(2\right)}∼e^{+\bar{t}},$ which, however, do not coincide with the original direct-time trajectories. Note that symmetric time reversal in conjunction with swapping $x^{\left(1\right)}$ and $x^{\left(2\right)}$ matches the original trajectories.

## 3. The Random Bridge Models

Our analysis requires the presence of *true randomness* as without antecedent causality or additional assumptions, deterministic equations are time-symmetric and fail to establish an arrow of time. Without effective randomisation, deterministic equations tend to force a choice between initial and final conditions, implicitly introducing antecedent causality and bringing us back to the logical circle discussed in the introduction. Incorporating true randomness into the model is crucial: a model without random fluctuations (or at least some form of emergent stochasticity, such as chaotisation associated with dynamical mixing) is rigid and could not satisfy both the initial and final conditions at the same time. *Determinism* would force us to choose between the initial conditions set, say, at $t=t_{1}$ and final conditions, say, set at $t=t_{2}>t_{1}$, making the model time-directional. Having preference for initial conditions introduces discrimination of the directions of time and is a de factor implementation of thinking associated with antecedent causality. Randomness provides a natural justification for imposing time-symmetric temporal boundary conditions

(11) $$x\left(t_{1}\right)=x_{1},\;\;\;\;x\left(t_{2}\right)=x_{2}$$

which are commonly referred to as *stochastic (random) bridges*. The associated probability distribution f=f(x,t) then satisfies the corresponding initial and final conditions

(12) $${\left(f\right)}_{t=t_{1}}=\delta \left(x-x_{1}\right),\;\;\;{\left(f\right)}_{t=t_{2}}=\delta \left(x-x_{2}\right)$$

This work considers two models for the laws of nature; both of these models involve true randomness generated by *Wiener processes* and, despite their diffusion character, possess some temporal symmetry. Due to the presence of randomness, these models are inevitably related to some forms of the *Fokker–Planck equation*. In this context, two types of time reversal (or reversibility) have been considered: *symmetric* following Kolmogorov [17] and *antisymmetric* following Anderson [18].

**Figure 2:**
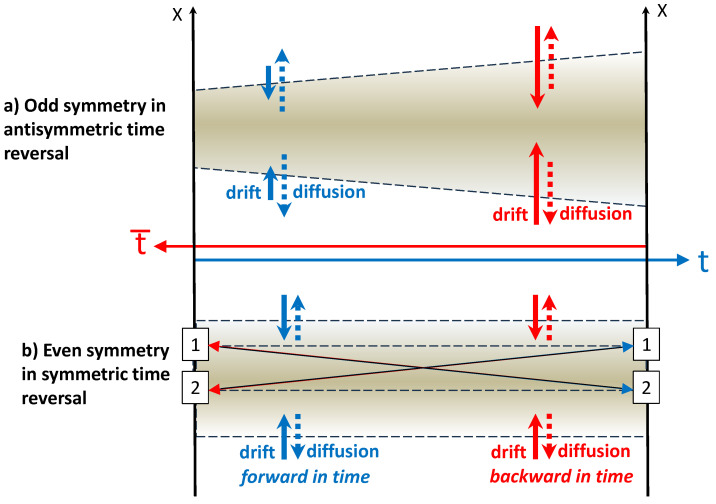
Illustration of symmetric and antisymmetric time reversals in stochastic systems. The shaded areas reflect probability densities; the dotted and solid arrows indicate the magnitudes of the diffusion and drift, respectively, for the forward (blue) and reversed (red) processes. The long arrows illustrate transition probabilities between states 1 and 2, which are consistent with detailed balance required by Kolmogorov’s reversibility conditions.

Kolmogorov’s symmetric time reversal leaves the governing equations formally unchanged when expressed in the new time variable $\bar{t}$, even though this re-labelling does not ensure that the system’s evolution is literally reversed, but as discussed previously, the direct and reverse evolutions are evenly similar. In contrast, Anderson’s antisymmetric time reversal focuses on the actual behaviour of the system—when time is reversed, the system retraces its exact steps, visiting each state in the reverse order. Symmetric time reversal emphasises the formal invariance of the laws under a change of the time coordinate, whereas antisymmetric time reversal highlights that the dynamics themselves are truly reversible, with forward and backward trajectories matching. As discussed in the Appendix A and Appendix B, matching random trajectories forward and backward in time requires to use the symmetrised *Stratonovich* interpretation [19] of stochastic equations instead of a more common interpretation due to *Ito* [20]. Because Kolmogorov’s notion of reversibility is fundamentally a conceptual statement about the symmetry of the transition probabilities (i.e., detailed balance), it is not tied to any specific interpretation of stochastic differential equations and requires only consistent definitions forward and backward in time. In other words, Kolmogorov’s reversibility does not directly reverse stochastic trajectories and does not specifically require the symmetrised formulation provided by the Stratonovich integral. It simply takes a direct-time model and applies the same model backward in time; therefore, this model can be equivalently expressed with any formulation of the stochastic differential equations, and Ito’s formulation tends to be more convenient. The two approaches to reversing time result in the corresponding models derived in the Appendix A and Appendix B, schematically illustrated in Figure 2 and presented below.

### 3.1. Markov Bridge Model with Odd Temporal Symmetry

The true randomness is conventionally represented by a stochastic *Markov diffusion process* $x_{t}=x\left(t\right)$, governed by a system of *Stratanovich stochastic differential equations* (SDEs), as discussed in the Appendix A and Appendix B. This process is considered within the time interval $t\in [t_{1},t_{2}]$ subject to initial and final boundary conditions.

The probability distribution function is factorised f(x,t)=φ(x,t)ψ(x,t)/C. Under conditions specified below, the functions φ(x,t) and ψ(x,t) satisfy the corresponding direct-time and reverse-time Fokker–Planck (Kolmogorov forward) equations

(13) $$\frac{\partial \varphi }{\partial t}=-\frac{\partial a^{i}\varphi }{\partial x^{i}}+\frac{\partial }{\partial x^{i}}\left(B^{ij}\frac{\partial \varphi }{\partial x^{j}}\right),\;\;\mathrm{with}\;\;{\left(\varphi \right)}_{t=t_{1}}=\delta \left(x-x_{1}\right)\;$$

(14) $$\frac{\partial \psi }{\partial t}=-\frac{\partial a^{i}\psi }{\partial x^{i}}-\frac{\partial }{\partial x^{i}}\left(B^{ij}\frac{\partial \psi }{\partial x^{j}}\right),\;\;\mathrm{with}\;\;{\left(\psi \right)}_{t=t_{2}}=\delta \left(x-x_{2}\right)$$

These equations—see Appendix A—exhibit an antisymmetric correspondence of the coefficients characterising the respective direct-time and reverse-time stochastic processes. Here, $t_{1}\leq t\leq t_{2}$ and the parameters of the model satisfy the following conditions ensuring odd symmetry

(15) $$\frac{\partial a^{i}}{\partial x^{i}}=0,\;\;B^{ij}=\frac{b^{ik}b^{jk}}{2}=B^{ji},\;\;\frac{\partial b^{ik}}{\partial x^{i}}=0$$

As stated in Proposition A1, these constrains ensure that the formulation of the problem is exactly odd-symmetric: when time is reversed, the model still specifies the same random process and no preference for the direction of time is introduced by the model. Note that the diffusion matrix $B^{ij}$ is symmetric and positive semidefinite and the drift coefficients $a^{i}$ are Stratanovich drifts, not Ito drifts. Under odd-symmetric conditions, the diffusion coefficients can be relatively small (and, as discussed in Section 2, are physically expected to be small). Constraints (15) are both physical and mathematical. From a physical perspective, the constraint $\partial a^{i}/\partial x^{i}=0$ preserves the phase volumes and entropy while $\partial b^{ik}/\partial x^{i}=0$ upholds the well-mixed conditions. From a mathematical perspective, constraints (15) ensure that the direct-time and reversed-time processes are equivalent despite evolving in the opposite directions in time. This odd symmetry of time reversal is not trivial: as time reverses, the diffusion and diffusion-induced drift terms change sign while the sign of the principal drift $a^{i}\left(x,t\right)$ remains the same in (13) and (14). The conventional (Ito) formulation of SDE becomes time-directional when $b^{ik}=$ $b^{ik}\left(x,t\right)$ depends on x and is not time-symmetric—time reversal of stochastic trajectories generally requires Stratanovich formulation of SDEs. Therefore, these are Stratanovich drifts $a^{i}$ and not Ito drifts $A^{i}$ that are deemed to represent deterministic physical quantities.

The normalisation constant *C* is determined by $C=\varphi \left(x_{2},t_{2}\right)=\psi \left(x_{1},t_{1}\right)$ so that the normalisation of *f*

(16) $$\int fdx=1,\;\;\;\;\;f=\frac{\phi \psi }{C}$$

is preserved by the model, since Equations (13) and (14) imply that equation for *f* has a conservative form

(17) $$\frac{\partial f}{\partial t}+\frac{\partial a^{i}f}{\partial x^{i}}=\frac{\partial }{\partial x^{i}}\left(B^{ij}f\left(\frac{\partial \ln\varphi }{\partial x^{j}}-\frac{\partial \ln\psi }{\partial x^{j}}\right)\right)$$

The model allows us to introduce two entropies, i.e., the direct-time entropy $S_{\varphi }$ and reverse-time entropy $S_{\psi }$:

(18) $$S_{\varphi }\left(t\right)=\int \varphi \ln\left(\frac{e}{\varphi }\right)dx,\;\;\;S_{\psi }\left(t\right)=\int \psi \ln\left(\frac{e}{\psi }\right)dx$$

It is easy to check that the direct-time entropy cannot decrease in time

(19) $$\frac{dS_{\varphi }}{dt}=-\int \frac{\partial \varphi }{\partial t}\ln\left(\varphi \right)dx=\underset{=0}{\underset{︸}{\int \varphi \frac{\partial a^{i}}{\partial x^{i}}dx}}+\underset{\Theta_{\varphi }}{\underset{︸}{\int \frac{B^{ij}}{\varphi }\frac{\partial \varphi }{\partial x^{i}}\frac{\partial \varphi }{\partial x^{j}}dx}}=\Theta_{\varphi }\geq 0$$

and that the reverse-time entropy cannot increase in time

(20) $$\frac{dS_{\psi }}{dt}=-\int \frac{\partial \psi }{\partial t}\ln\left(\psi \right)dx=\underset{=0}{\underset{︸}{\int \psi \frac{\partial a^{i}}{\partial x^{i}}dx}}-\underset{\Theta_{\psi }}{\underset{︸}{\int \frac{B^{ij}}{\psi }\frac{\partial \psi }{\partial x^{i}}\frac{\partial \psi }{\partial x^{j}}dx}}=-\Theta_{\psi }\leq 0$$

Conditions (8) are essential for these conclusions. In evaluation of the integrals by parts, we assume that that domain either infinite or, if it is bounded, does not allow any probability fluxes through its boundaries, that is, the components of the drift and diffusion flux normal to the boundary vanish. Assuming that $B^{ij}$ projection into the domain under consideration is non-degenerate (and therefore, positively defined), we note that $dS_{\varphi }/dt=0$ can be only in a steady-state solution φ=const. Similarly, $dS_{\psi }/dt=0$ is only when ψ=const. Hence, the equations admit a unique steady-state solution that is constant, which can only be realised within domains of finite measure due to the preserved normalisation of the distribution functions.

Let us assume that such a domain corresponds to an isolated state of a subsystem and lies on the associated manifold, whose dimension is determined by the number of free degrees of freedom of the subsystem. This domain is termed *ergodically isolated* if neither dynamical trajectories—assumed to satisfy the ergodicity conditions—nor diffusive fluxes can cross its boundaries or escape the domain. Since the domain lies on a manifold of reduced dimension, the condition of ergodic isolation implies that there is no diffusion flux across the manifold. Consequently, the diffusion matrix is degenerate, allowing diffusive fluxes along the manifold but not across it. As noted above, the projection of $B^{ij}$ onto the domain manifold is assumed to be non-degenerate. Given that homogeneous Hamiltonian systems conserve energy, an ergodically isolated domain represents an invariant manifold corresponding to a constant-energy surface.

Since entropy is a monotonic and, in case of a finite volume, bounded function of time, entropy monotonically converges to its maximal value $S\left(t\right)\to S_{\max}=\ln\left(V\right)$ in a finite measure (surface volume) V, the distributions φ or ψ should converge (as measured by the corresponding *Kullback–Leibler divergency*) to constant stationary solutions forward in time for φ and backward in time for ψ.

Under the conditions specified above, this behaviour of entropy can be summarised by the following proposition.

**Proposition** **1.** *The odd-symmetric (antisymmetric) model has two entropies: direct-time entropy $S_{\varphi }$ and reverse-time entropy $S_{\psi }$, which monotonically increase forward in time and backward in time, respectively, until they stabilise upon reaching steady-state solutions. These solutions are necessarily constant and can only be achieved in finite-measure domains.*

Identifying these distinct entropies $S_{\varphi }$ and $S_{\psi }$ might be difficult as only the overall distribution *f*∼φψ is directly measurable. Hence, we may wish to define a joint entropy

(21) $$S_{f}\left(t\right)=\int f\ln\left(\frac{e}{f}\right)dx=\underset{S_{f|\psi }}{\underset{︸}{\int \frac{\psi }{C}\varphi \ln\left(\frac{1}{\varphi }\right)dx}}+\underset{S_{\psi |\varphi }}{\underset{︸}{\int \frac{\varphi }{C}\psi \ln\left(\frac{1}{\psi }\right)dx}}+\ln\left(eC\right)$$

which represents a sum of the Kullback–Leibler divergencies $S_{f|\psi }$ and $S_{f|\varphi }$, and may increase or decrease in time according to

(22) $$\begin{array}{cc} \frac{dS_{f}}{dt} & =-\int \frac{\partial f}{\partial t}\ln\left(f\right)dx=\underset{=0}{\underset{︸}{\int f\frac{\partial a^{i}}{\partial x^{i}}dx}}+\int B^{ij}\frac{\partial f}{\partial x^{i}}\left(\frac{\partial \ln\varphi }{\partial x^{j}}-\frac{\partial \ln\psi }{\partial x^{j}}\right)dx\\ \; & =\underset{\Theta_{f|\psi }}{\underset{︸}{\int \frac{\psi }{C}\frac{B^{ij}}{\varphi }\frac{\partial \varphi }{\partial x^{i}}\frac{\partial \varphi }{\partial x^{j}}dx}}-\underset{\Theta_{f|\varphi }}{\underset{︸}{\int \frac{\varphi }{C}\frac{B^{ij}}{\psi }\frac{\partial \psi }{\partial x^{j}}\frac{\partial \psi }{\partial x^{i}}dx}}=\Theta_{f|\psi }-\Theta_{f|\varphi } \end{array}$$

The dissipation terms $\Theta_{f|\psi }\geq 0$ and $\Theta_{f|\varphi }\geq 0$ are related to the corresponding dissipation terms $\Theta_{\varphi }$ and $\Theta_{\psi }$ and vanish when the corresponding terms also vanish, so that $\Theta_{f|\psi }=0$ corresponds to $\Theta_{\varphi }=0$ and $\Theta_{f|\varphi }=0$ corresponds to $\Theta_{\psi }=0$.

The model (13)–(17) is consistent with dynamic constraint (8), which are inherited from fundamental mechanical models and preserves the phase volume, as well as converging to uniform distributions in the phase space (or to equidistribution between quantum states) when and if thermodynamic equilibrium is achieved for a subsystem kept in an isolated state in a domain of a finite measure. Under ergodically isolated conditions specified above and assumed valid in the rest of the paper, an isolated subsystem should preserve its energy *H*, which corresponds exactly to microcanonical distribution in statistical physics, implying equidistribution between all available states, whose number is proportional to the phase volume. The unusual feature of the bridge model is presence of two entropies $S_{\varphi }$ and $S_{\psi },$ which evolve in the opposite directions. This is a key feature of the model, which is discussed in the next section.

### 3.2. Markov Bridge Model with Even Temporal Symmetry

As detailed in Appendix B, the Kolmogorov’s theory of even-symmetric temporal reversibility of stationary Markov diffusion processes can be represented by modified Fokker–Planck equations governing functions ϕ(x,t) and ψ(x,t)

(23) $$\eta \frac{\partial \phi }{\partial t}=\frac{\partial }{\partial x^{i}}\left(\eta B^{ij}\frac{\partial \phi }{\partial x^{j}}\right),\;\;\mathrm{with}\;\;{\left(\phi \right)}_{t=t_{1}}=\delta \left(x-x_{1}\right)$$

(24) $$-\eta \frac{\partial \psi }{\partial t}=\frac{\partial }{\partial x^{i}}\left(\eta B^{ij}\frac{\partial \psi }{\partial x^{j}}\right),\;\;\mathrm{with}\;\;{\left(\psi \right)}_{t=t_{2}}=\delta \left(x-x_{2}\right)$$

where $t_{1}\leq t\leq t_{2}$ and $\phi \left(x,t;x_{1},t_{1}\right)=\eta \left(x_{1}\right)\varphi \left(x,t;x_{1},t_{1}\right)/\eta \left(x\right)$. Assuming $A^{i}=A^{i}\left(x\right)$ and $B^{ij}=B^{ij}\left(x\right)$, Kolmogorov [17] established the following condition for reversibility of the process

(25) $$A^{i}\eta =\frac{\partial B^{ij}\eta }{\partial x^{j}}\;⟹{\tilde{A}}^{i}=B^{ij}\frac{\partial \ln\eta }{\partial x^{j}}$$

where

(26) $${\tilde{A}}^{i}=A^{i}-\frac{\partial B^{ij}}{\partial x^{j}}=a^{i}-\frac{b^{ik}}{2}\frac{\partial b^{jk}}{\partial x^{j}},\;\;\;B^{ij}=\frac{b^{ik}b^{jk}}{2}=B^{ji},\;\;\;A^{i}=a^{i}+\frac{b^{jk}}{2}\frac{\partial b^{ik}}{\partial x^{j}}$$

and η=η(x) is a steady-state distribution that must exist. In even-symmetric models, the diffusion $B^{ij}$ and drift $A^{i}$ coefficients have similar magnitudes. The overall distribution *f* is normalised with the normalisation constant $C_{1}$ and governed by

(27) $$f=\frac{\eta \phi \psi }{C_{1}},\;\;\;\frac{\partial f}{\partial t}=\frac{\partial }{\partial x^{i}}\left(B^{ij}f\left(\frac{\partial \ln\phi }{\partial x^{j}}-\frac{\partial \ln\psi }{\partial x^{j}}\right)\right)$$

The direct-time and reverse-time entropies are defined by

(28) $$S_{\phi }\left(t\right)=\int \eta \phi \ln\left(\frac{e}{\phi }\right)dx,\;\;\;S_{\psi }\left(t\right)=\int \eta \psi \ln\left(\frac{e}{\psi }\right)dx$$

and display monotonic behaviours

(29) $$\frac{\partial S_{\phi }}{\partial t}=-\int \eta \frac{\partial \phi }{\partial t}\ln\left(\phi \right)dx=\int \frac{\eta }{\phi }B^{ij}\frac{\partial \phi }{\partial x^{i}}\frac{\partial \phi }{\partial x^{j}}dx=\Theta_{\phi }\geq 0$$

(30) $$\frac{\partial S_{\psi }}{\partial t}=-\int \eta \frac{\partial \psi }{\partial t}\ln\left(\psi \right)dx=-\int \frac{\eta }{\psi }B^{ij}\frac{\partial \psi }{\partial x^{i}}\frac{\partial \psi }{\partial x^{j}}=-\Theta_{\psi }\leq 0$$

expected for proper entropies. The overall entropy

(31) $$S_{f}\left(t\right)=\int f\ln\left(\frac{e\eta }{f}\right)dx=\underset{S_{f|\psi }}{\underset{︸}{\int \eta \frac{\psi }{C_{1}}\phi \ln\left(\frac{1}{\phi }\right)dx}}+\underset{S_{f|\phi }}{\underset{︸}{\int \eta \frac{\phi }{C_{1}}\psi \ln\left(\frac{1}{\psi }\right)dx}}+\ln\left(eC_{1}\right)$$

changes consistently with partial entropies

(32) $$\begin{array}{cc} \frac{dS_{f}}{dt} & =-\int \frac{\partial f}{\partial t}\ln\left(\frac{f}{\eta }\right)dx=\int \frac{1}{\phi \psi }\frac{\partial (\phi \psi )}{\partial x^{i}}\frac{\eta }{C_{1}}B^{ij}\left(\psi \frac{\partial \phi }{\partial x^{j}}-\phi \frac{\partial \psi }{\partial x^{j}}\right)dx\\ \; & =\underset{\Theta_{f|\psi }}{\underset{︸}{\int \frac{\eta }{C_{1}}\frac{\psi }{\phi }B^{ij}\frac{\partial \phi }{\partial x^{i}}\frac{\partial \phi }{\partial x^{j}}dx}}-\underset{\Theta_{f|\phi }}{\underset{︸}{\int \frac{\eta }{C_{1}}\frac{\phi }{\psi }B^{ij}\frac{\partial \psi }{\partial x^{j}}\frac{\partial \psi }{\partial x^{i}}dx}}=\Theta_{f|\psi }-\Theta_{f|\phi } \end{array}$$

so that $\Theta_{f|\psi }\geq 0$ and $\Theta_{f|\phi }\geq 0$ are consistent with the corresponding dissipations $\Theta_{\phi }\geq 0$ and $\Theta_{\psi }\geq 0$. As in the previous subsection, monotonic properties of the entropies ensure convergence to constant values of ϕ(x,t) forward in time and ψ(x,t) backward in time so that the overall distribution becomes stationary f(x,t)=η(x). Our analysis indicates that the following proposition holds.

**Proposition** **2.** *The even-symmetric model has two entropies: direct-time entropy $S_{\phi }$ and reverse-time entropy $S_{\psi }$, which are introduced as the corresponding Kullback–Leibler divergences from the x-dependent steady-state solution η=η(x) monotonically increase forward in time for $\;S_{\phi }$ and backward in time for $S_{\psi }$ until they stabilise upon reaching the steady-state solution.*

The first question is whether a model with true randomness can be both even-symmetric and odd-symmetric simultaneously. This requires that η=const and, therefore, ${\tilde{A}}^{i}$ and $a^{i}$ must vanish in (25) and (26) due to (15). Hence, possessing both symmetries is possible only in trivial cases, which are not of interest here. Brownian bridge is an example of a trivial case that possesses both symmetries, odd and even. When considering general laws of nature, the requirements for both types of symmetry become opposing: the potential character of ${\tilde{A}}^{i}$ in (25) and solenoidal character of $a^{i}$ in (15). Both symmetries (of the odd and even types) can coexist only in trivial cases or when applied to different degrees of freedom associated with effectively independent subsystems. If a system is isolated, then non-constant η(x) is not consistent with the microcanonical distribution and the observed laws of conventional thermodynamics. It seems, however, that non-uniform equilibrium distributions associated with the symmetric time reversal might be relevant to thermodynamic systems placed in strong gravity (such as stars and black holes).

Our consideration can now be summarised by the following proposition.

**Proposition** **3.** *With the exception of trivial cases or applications to distinct autonomous subsystems, the laws of nature involving true randomness cannot produce models that are simultaneously symmetric and antisymmetric, i.e., that exhibit both even and odd temporal symmetries. The antisymmetric model aligns well with conventional thermodynamics and observed reality and must, therefore, be dominant. The even-symmetric model might be relevant to autonomous systems with strong gravitational effects.*

## 4. Time-Symmetric Models and Their Relevance to the Observed Universe

While we consider two types of time-symmetric models in this section, the system of Equations (13) and (14), which constitutes an antisymmetric (or odd-symmetric) model, is of special interest here, as this model is consistent with thermodynamic reality. In this model, the deterministic component embodies the time-reversible dynamics of every minute particle in nature implementing principal physical laws, while the random component captures irreversible effects that drive changes in entropy.

This model exhibits temporal symmetry—specifically of the odd or antisymmetric type—and, crucially, has time-symmetric temporal boundary conditions. It also permits an explicit dependence on time, which may indicate global expansion affecting the space-time metric or other fundamental changes in the governing laws (assuming *t* represents some kind of a synchronised time).

### 4.1. The Role of the Final Conditions in Time-Symmetric Models

In our approach, we consciously avoid the conventional pitfall of granting unqualified primacy to initial conditions, which would inevitably imply the presence of antecedent causality—a concept that may hold relevance but which we prefer not to accept as an unconditional postulate, leaving room for it to emerge as a derived property within the framework.

The initial conditions at $t=t_{1}$ reflect the state of low entropy in the early Universe, which is fundamental for understanding irreversible thermodynamic behaviour driving the system towards equilibrium. This state may or may not be related to the Big Bang theory; however, if it is, the key point for us is that it was a low-entropy, well-ordered Big Bang. While the concept of low-entropy beginnings has been widely accepted and discussed since Boltzmann’s time, the final conditions are rarely mentioned. This omission corresponds to conventional thinking associated with antecedent causality.

In this work, we follow Schulman [13,15] and impose the final conditions at $t=t_{2}$ to mirror the initial conditions at $t=t_{1}$, not because the final conditions are known or knowable, but to avoid introducing time directionality and maintain symmetry in our formulation. Therefore, we set the final conditions to be the same as, or similar to, the initial conditions, assuming that the properties observed in the present are not greatly affected by the precise details of these final conditions. In simple terms, if we know nothing about the final conditions and do not wish to discriminate between the directions of time a priori, we must assume low-entropy final conditions to avoid building an a priori bias into our theories of nature. Suggestions that the cosmological final state of the Universe might be similar to its initial state have been repeatedly discussed in publications [11,12,13,14].

### 4.2. The Arrow of Time and Origin of Causality

If both the initial and final conditions are set, the entropy is minimal at the beginning, $t=t_{1}$, reaches its maximum midway at $(t_{1}+t_{2})/2$ (assuming full temporal symmetry), and decreases again at $t=t_{2}$ to its initial value. Clearly, we are located somewhere at a time *t* close to the origin, meaning $t-t_{1}\ll t_{2}-t$, and we are influenced by the initial conditions incomparably more strongly than by the final conditions. In the representation f=φψ/C (assuming odd symmetry), the function φ evolves actively forward in time according to the Fokker–Planck equation and the fundamental laws of the Universe, thereby increasing the entropy $S_{\varphi }$. In contrast, ψ remains nearly constant everywhere, and consequently, the reverse-time entropy $S_{\psi }$ is also nearly constant so that the joint entropy $S_{f}∼S_{\varphi }$ increases in time. We observe non-equilibrium states induced by preselection effect of the initial conditions, enabling us to make inferences about the early states of the Universe. However, we do not observe any postselection associated with the final conditions and have no certain knowledge about final conditions. The potential for the entropy $S_{\varphi }$ to increase further suggests substantial direct-time availability, whereas the reverse-time availability is almost entirely absent.

Despite our time-symmetric approach, the conditions we describe are practically indistinguishable from conventional interpretations. Suppose we aim to solve the problem within the time interval $\left[t_{1}^{\prime },t_{2}^{\prime }\right]$, where $t_{1}<t_{1}^{\prime }<t_{2}^{\prime }\ll t_{2}$. We can set ψ=const and f∼φ in (17), impose the initial conditions at $t=t_{1}^{\prime }$, and solve the Fokker–Planck equation in the form (17) forward in time from $t_{1}^{\prime }$ to $t_{2}^{\prime }$. No final conditions are required. However, if we attempt to specify the final conditions at $t=t_{2}^{\prime }$ instead of the initial conditions, we face significant difficulties due to the ill-posed nature of the problem. Consequently, we do not postulate antecedent causality but derive its most crucial implication—our preference for setting initial conditions rather than final conditions. Intuitively, we justify this principle by observing that it is the past that influences the future, not vice versa.

The main point is now summarised in the following proposition.

**Proposition** **4.** *The odd-symmetric interpretation of the laws governing the Universe, combined with time-symmetric temporal boundary conditions without postulating antecedent causality, gives rise to monotonic entropy growth, the arrow of time, and the observable emergence of temporal causal order as long as we are closer to the onset than to the end, i.e., $t_{1}<t\ll t_{2}.$*

This proposition, which suggests the existence of a thermodynamic arrow of time directed forward when $t_{1}<t\ll t_{2}$ and backward when $t_{1}\ll t<t_{2},$ overlaps with the conclusions deduced from Schulman’s model [13].

### 4.3. Thermodynamic Equilibrium in Odd-Symmetric Models

The thermodynamic behaviour is closely linked to the concept of equilibrium. The reverse-time entropy and associated reverse-time thermodynamics are at or near the global equilibrium state, whereas the direct-time entropy and associated direct-time thermodynamics are not. Nevertheless, local or partial equilibria are commonly achieved through direct-time evolution processes.

Following Penrose [21], let us imagine the Universe as a maze of constraints within a space of very high dimension, maintained by the deterministic laws governing the Universe. The random component is represented by diffusion. Direct-time evolution begins in a region of small volume and progresses towards larger and larger volumes, increasing entropy $S_{\varphi }$. Within certain cells of the maze, equilibrium may be reached, implying a uniform distribution within the cell but not between cells, meaning the system remains far from global equilibrium.

More formally, a subsystem may become isolated within a time interval $\left[t_{1}^{\prime },t_{2}^{\prime }\right]$, where $t_{1}<t_{1}^{\prime }<t_{2}^{\prime }\ll t_{2}$, and thus, preserves its total energy. For the degrees of freedom associated with this subsystem, the Fokker–Planck Equation (13) evolves towards uniform distributions f∼φ=const, representing microcanonical equilibrium, while ψ∼consteverywhere within the interval. Such equilibrium may later be disturbed by coupling the subsystem to others, continuing the direct-time evolution. The reverse-time evolution remains negligible, as ψ is very close to constant throughout the accessible region.

### 4.4. Distinctive Features of the Even-Symmetric Models

While our previous consideration focusses on the antisymmetric model, it is worthwhile to explore the implications of the second model with even temporal symmetry. In many respects, the symmetric model behaves similarly to the antisymmetric model, but its equilibrium becomes a joint property of direct-time and reverse-time evolutions (rather than their more autonomous properties, as in the antisymmetric model), implying a stronger conceptual coupling of the direct-time and reverse-time submodels. This equilibrium, f=η(x), is significantly non-uniform, while a non-equilibrium state is expressed as the product f∼ηϕψ involving two multiplicative functions: ϕ, evolving forward in time, and ψ, evolving backward in time.

Considering that distributions in phase spaces of very large dimension are usually sharp, only regions where $\eta \left(x\right)\approx \eta_{\max}$ are statistically significant, effectively reducing the entire space to the vicinity of a single point or a relatively small set of points where $\eta \left(x_{\max}\right)=\eta_{\max}$. Despite this highly localised state, where ϕ∼ψ∼const and f∼η(x), the entropy $S_{f}$ reaches its maximum. This behaviour, although unusual from a conventional thermodynamic perspective, is indeed observed in nature. For example, black holes exhibit a very limited set of defining parameters but have the highest possible entropy. Here, we suggest only that the even type of temporal symmetry may be relevant to black holes and, of course, not that Equations (23) and (24) specifically govern them.

Another key feature of the symmetric model is strong dissipation. Although random effects are often assumed to be small, allowing deterministic equations to remain reasonably accurate, there are mechanisms of amplification of the diffusion effects due to dynamic mixing. In the symmetric model, there is an additional amplification mechanism associated with strong convergence of the drift producing sharp gradients and with ensuring that deterministic and random terms remain of the same order. This intensifies dissipation, and for a given characteristic energy *E*, results in high entropy changes ΔS, implying very low temperatures, T∼E/ΔS. Once again, such unusual behaviour is conventionally associated with black holes.

## 5. The Effects Associated with the Reverse-Time Part of the Model

Despite maintaining temporal symmetry, the behaviour discussed in the previous section is indistinguishable from conventional entropy-increasing evolution, while the final conditions remain hidden. This section aims to explore potential effects associated with the model’s reverse-time dynamics. To observe such effects, one might consider investigating the remote future $t_{1}\ll t<t_{2}$, This, however, is of limited interest, as thermodynamic behaviour in the distant future would closely mirror the present, with the arrow of time simply reversed.

At this point, it is important to clarify that the influence of the initial boundary conditions on the random terms generally remains indefinite. When constrained in the past, these random components increasingly propagate uncertainty into the future, a behaviour that corresponds to the direct parabolic terms in the Fokker–Planck equation. While the relative magnitude of these terms may diminish, their fundamental nature remains unchanged, consistently reflecting direct-time physics. These direct parabolic terms do not gradually transform into reverse-parabolic terms; rather, the direct-parabolic and reverse-parabolic components coexist, competing with one another. A similar consideration applies to the final conditions—their influence does not entirely vanish even during early stages. Although the relative magnitude of these effects may be small, they could, in principle, be detected, albeit with extreme—if not insurmountable—difficulty in practice.

Accordingly, the principal conceptual question is how the reverse-time component of the antisymmetric model could manifest in present conditions, no matter how subtle or insignificant these effects might be. Schulman [13] suggested that both direct-time and reverse-time effects may coexist. Tamm [14] proposed that the future state of the Universe could influence its current rate of expansion—this is a related but distinct issue concerning the deterministic dynamics of the Universe. In contrast, our analysis focuses on detecting subtle effects associated with randomness and linked to the postselection of the final conditions. Specifically, we seek to understand how such effects might appear to an observer when direct-time evolution remains dominant and preserves a strong conventional arrow of time. There are two cases of interest: emergence of a small global gradient of function ψ and a local variation of ψ in a tiny isolated system, while ψ remains (nearly) constant everywhere.

### 5.1. Detecting Postselection

Let us assume that, although ψ is close to a constant, there exists a small global gradient of ψ, while φ evolves intensively forward in time. First of all, the presence of a global gradient in ψ affects only random processes and has no influence on deterministic dynamics. Therefore, our fundamental mechanical laws remain unchanged. The existence of a gradient in ψ implies that the postselection of random trajectories by the final temporal boundary conditions introduces some preferences or biases. For example, postselection could, at least in principle, create a slight preference for heads over tails. If so, tossing a perfectly fair coin many times would reveal a tiny bias towards heads—provided that we could detect even the smallest bias, which is doubtful since a physical coin is unlikely to behave ideally. Another issue is why the final conditions would favour heads. If the toss outcomes—heads or tails—form a direction in phase space that is perpendicular to the gradient of ψ, then the final conditions imposed on the Universe would have no effect on the coin’s outcome, which, practically, is the most likely scenario.

A more precise set of experiments can be conducted by measuring quantum spin, assuming that, by default, spin up $|↑\rangle$ and spin down $|↓\rangle$ are equally probable outcomes. Theoretically, postselection can introduce certain biases. Our particle may inadvertently be entangled with another particle elsewhere in the universe. This distant particle could be influenced by the final conditions, for instance, through the postselection of a particular magnetic field in the final stages of the universe. It goes without saying that detecting such an effect remains purely a hypothetical possibility.

Some may interpret the reverse-time evolution of ψ as introducing retrocausality. Even if we accept this perspective, conducting experiments to detect retrocausality is problematic, as the concept of causality, while useful, is rather vague and largely based on intuition. Moreover, the conventional “flow of causality”, associated with the evolution of φ, would overwhelm any other effects.

Consider a system that remains isolated during the time interval $[t_{1}^{\prime },t_{2}^{\prime }].$ Initially, at $t=t_{1}^{\prime }$, it is generally not in equilibrium (due to preselection by the low-entropy initial conditions imposed on the Universe). However, after some time $t=t_{1}^{\prime }+\Delta t$, the equilibrium distribution φ=const is established. Similarly, postselection could be detected only after $t=t_{2}^{\prime }-\Delta t$, as ψ would reach equilibrium ψ=const backwards in time when $t<t_{2}^{\prime }-\Delta t$. In practice, an experimentalist would observe an increase in thermodynamic fluctuations just before opening the system to the outside world. 

Our Universe has existed for 13 billion years and is still far from equilibrium. This suggests that, if final conditions do exist, they must lie far beyond 13 billion years into the future. It is, therefore, highly likely that these conditions have no appreciable postselection effect at present. Even if they exerted some minor influence, detecting postselection would remain challenging.

### 5.2. Observing Localised Antisystems

The direct-time and reverse-time components seem to be linear and independent, coupled only through the joint probability distribution f=φψ/C. However, this apparent independence is misleading and typically arises when Liouville-type equations are applied in spaces of extremely high dimension. As soon as we attempt to reduce the number of variables to a practically justifiable level, the subsystems exhibit strong non-linear interactions, as illustrated in the following example.

Consider a small system that is completely isolated—this system reaches internal equilibrium $\varphi_{s}=\mathrm{const}$ and can, in principle, exist in this state indefinitely, even if it is not in equilibrium with the universe, i.e., $\varphi_{s}\neq \varphi_{u}$. Similarly, an isolated *antisystem* with constant $\psi_{a}\neq \psi_{u}$ could, at least in principle, exist in our time. While such systems and antisystems can exist theoretically, preserving their properties in a hostile environment over long durations seems problematic. However, another possibility arises from the stochastic nature of randomness, reflected in the presence of fluctuations. Therefore, tiny systems and antisystems that are not in equilibrium with their environments may occasionally appear (and disappear) due to fluctuations, provided that such appearances comply with the deterministic laws of the Universe.

What properties would such a tiny antisystem exhibit if it appeared around us? Our world is characterised by varying φ=φ(x,t) and nearly constant $\psi =\psi_{u}=\mathrm{const}$, whereas the antisystem has $\psi_{a}>\psi_{u}$. Since the antisystem is small, the global laws of thermodynamics should remain valid as long as its behaviour can be described in conventional thermodynamic terms. On the surface, this seems problematic since $S_{\psi }$ tends to decrease in time, behaving unconventionally. However, instead of the symmetric entropy $S_{f}$, which behaves inconsistently within our thermodynamic framework, we can define an effective or antisymmetric entropy:

(33) $$S_{\mathrm{eff}}=S_{\varphi }-S_{\psi }$$

which remains monotonic in time and cannot decrease, i.e., $dS_{\mathrm{eff}}/dt\geq 0$. With this definition of entropy, the conventional laws of thermodynamics remain intact.

Let us consider the effect of energy exchange δQ from a small antisystem with $\psi_{a}>\psi_{u}$ and $\varphi_{a}=\varphi_{s}$ to a system with $\varphi_{s}$ and $\psi_{s}=\psi_{u}$ so that the system and antisystem energies become after the exchange $E_{s}+\delta Q$ and $E_{a}-\delta Q$ respectively. Considering autonomous, intrinsic properties of these systems, the entropy variation due to energy change can be expressed as:

(34) $$dS_{s}=\frac{dE_{s}}{T_{s}},\;dS_{a}=\frac{dE_{a}}{T_{a}}$$

where the energies, entropies, and temperatures are the corresponding intrinsic properties of the system and antisystem. Since $dE_{s}=\delta Q$ and $dE_{a}=-\delta Q$, we obtain:

(35) $$dS_{\mathrm{eff}}=dS_{s}-dS_{a}=\left(\frac{1}{T_{s}}+\frac{1}{T_{a}}\right)\delta Q\geq 0.$$

Unlike in conventional thermodynamic interactions, where $dS=\left(T_{s1}^{-1}-T_{s2}^{-1}\right)\delta Q$, the sign of dS depends on the temperatures of the interacting systems s1 and s2. In contrast, $dS_{\mathrm{eff}}$ is always positive, strongly favouring energy transfer from the antisystem to the system. This process does not terminate even if the intrinsic temperature of the antisystem becomes low, continuing until the antisystem loses all its energy. Note that a system cannot simultaneously possess both direct-time and reverse-time availabilities as this would be thermodynamically unstable due to immediate contact of the subsystems. While a specialised antisymmetric version of thermodynamics and kinetics has been developed for the interactions between thermodynamic systems and antisystems [22,23,24,25], our objective here is to highlight this unusual behaviour and determine whether it may manifest in the known Universe. The complete transfer of energy into the environment strongly resembles properties of antimatter in its interactions with matter. We may indeed have antisystems present in our world in the form of antiparticles which, according to Feynman, can be deemed to “travel” to us from the future. The theory introducing an antisymmetric extension of thermodynamics and kinetics from systems to antisystems is experimentally testable, even at the present level of technologyby [22,23,24,25].

The analysis of this section is summarised by the following proposition.

**Proposition** **5.** *The direct-time and reverse-time random effects, respectively characterised by φ and ψ, tend to persist indefinitely and conceptually coexist without blending or converting into one another, while remaining antagonistic from a thermodynamic perspective. The entities governed by these direct-time and reverse-time effects are respectively termed thermodynamic systems and antisystems. The practical detection of reverse-time effects remains problematic under present conditions, which are dominated by the direct arrow of time—unless compact thermodynamic antisystems can physically exist in our world, in which case such properties could, in principle, be examined experimentally. The fundamental question of whether antimatter possesses the properties of thermodynamic antisystems remains open.*

## 6. Conclusions

Our present analysis is based on the following assumptions:

1. Most of the *fundamental physical laws* governing nature (encompassing classical, relativistic, and quantum mechanics) are *deterministic* (implying quantum unitary determinism), time-reversible (i.e., they preserve entropy), and are close to being time-symmetric;
2. *Randomness* is inherently present in nature, as reflected in thermodynamics, statistical physics, and in some irreversible quantum effects;
3. The Universe is subject to *low-entropy initial conditions* in the distant past;
4. The Universe is also subject to some *low-entropy final conditions* in an even more distant future (i.e., the *Schulman condition*).

Some of these assumptions—specifically Assumptions 1 and 3—are more conventional. We explore the implications of adding Assumptions 2 and 4—omnipresent randomness and the Schulman condition—which remove the inherent asymmetry of time found in most conventional theories and allow us to demonstrate the *arrow of time* without postulating antecedent causality. The ubiquitous presence of randomness is responsible for time-directional effects and inevitably leads to *Fokker–Planck*-type equations in high-dimensional spaces. Further analysis, based on temporal symmetry, results in two principal types of models:

A. Antisymmetric (odd-symmetric) models, which characterise conventional thermodynamic behaviour;
B. Symmetric (even-symmetric) models, which may be associated with thermodynamic systems in strong gravitational fields.

Our analysis explains the emergence of the world as we know it, encompassing conventional thermodynamics, the arrow of time, and a form of antecedent causality—all of which are deduced rather than postulated. This behaviour, associated with the relatively early stages of the Universe’s evolution, arises from mathematical properties of *Markov bridges* and does not rely on common assumptions that impose a preferred direction of time.

Additionally, we explore the effects of reverse-time dynamics associated with final conditions, which are presumably far more remote than the initial conditions, and find that such effects would be difficult to detect in the real world—except through the emergence of antisystems whose properties closely resemble those of antimatter. Whether antimatter possesses the properties of thermodynamic antisystems is experimentally testable.

## Data Availability

The original contributions presented in this study are included in the article. Further inquiries can be directed to the corresponding author.

## Appendix A. Notes on Bridge Conditions in Markov Models

This Appendix overviews mathematical properties of *Markov diffusion bridges* that are used in the main text establishing conditions for invariant time reversal.

### Appendix A.1. Stochastic Equations Forward and Backward in Time

Consider a family of stochastic *Markov diffusion processes* $x_{t}$ that has the same semigroup generator coefficients $a^{i}\left(x_{t},t\right)$ and $b^{ik}\left(x_{t},t\right),$ and is governed by the following system of *Ito stochastic differential equations* (SDEs)

(A1) $$dx_{t}^{i}=A^{i}\left(x_{t},t\right)dt+b^{ik}\left(x_{t},t\right)dw_{t}^{k}$$

The default Einstein summation convention applies here and throughout the Paper, while $w_{t}^{k}$ denotes independent *Wiener processes*. In addition to the process (A1) defined forward in time, we can introduce the reversed time $\bar{t}=-t$ and run another process ${\bar{x}}_{\bar{t}}$ forward in time $\bar{t}$ and backward in time *t*, as specified by the stochastic differential equation

(A2) $$d{\bar{x}}_{\bar{t}}^{i}={\bar{A}}^{i}\left({\bar{x}}_{\bar{t}},\bar{t}\right)d\bar{t}+{\bar{b}}^{ik}\left({\bar{x}}_{\bar{t}},\bar{t}\right)d{\bar{w}}_{\bar{t}}^{k}$$

The drift and diffusion coefficients ${\bar{A}}^{i}$ and ${\bar{b}}^{ik}$ are generally different from $A^{i}$ and $b^{ik}$. While we are interested in cases and conditions under which the processes ${\bar{x}}_{\bar{t}}\left(-t\right)$ and $x_{t}\left(t\right)$ are equivalent, we do not imply yet at this stage any equivalence or similarity of ${\bar{x}}_{\bar{t}}$ and $x_{t}$ and treat these processes in (A1) and (A2) as being different. As demonstrated by Anderson [18], ${\bar{w}}_{\bar{t}}^{k}$ is related to $w_{t}^{k}$ but with some adjustments to preserve the retrocasual structure of the reverse-time stochastic integrals and ensure that ${\bar{x}}_{\bar{t}}\left(-t\right)=x_{t}\left(t\right)$ in a strong sense. Here, we just assume that ${\bar{w}}_{\bar{t}}^{k}$ is another equivalent Wiener process.

It should be noted that Ito calculus is time-directional enforcing independence of $x_{t}\left(t\right)$ of $w_{t}\left(t^{\prime }\right)$ when $t^{\prime }>t$. This understanding is implemented in the numerical definition of the product $b^{ik}\left(x_{t},t\right)dw_{t}^{k}$ in the corresponding stochastic integral, where $b^{ik}\left(x_{t},t\right)$ is evaluated using the value of $x_{t}$ at the beginning of each time step. A more general approach can be formally expressed as $b^{ik}{\left(x_{t},t\right)}_{\gamma }dw_{t}^{k}$, implying that $b^{ik}$ is evaluated as $b^{ik}\left(x_{t}+\gamma dx_{t},t\right)dw_{t}^{k},$ where 0≤γ≤1 and γ=0 corresponds to the Ito definition, γ=1/2 to the Stratanovich definition, and γ=1 to the anti-Ito definition of the stochastic integral and the corresponding differential equations. Note that, when time is reversed, the Ito definition is converted into the anti-Ito definition, rather than yielding the Ito definition evaluated backward in time, that is $\bar{\gamma }=1-\gamma$. Only the *Stratanovich formulation of SDEs*, which has $\bar{\gamma }=\gamma =1/2$ and is conventionally denoted by $b^{ik}\left(x_{t},t\right)\circ dw_{t}^{k},$ is time-symmetric, corresponding to the same Stratanovich formulation when evaluated backward in time. Note that while Stratanovich calculus removes some temporal asymmetry of the Ito calculus, it is still conceptually related to Ito calculus and, similar to Ito calculus, has some inherent directionality of time built-in into its foundation. The Stratanovich SDE that corresponds to (A1) is specified by the following equation with the new drift $a^{i}$ adjusted by removing diffusion-generated drift component from $A^{i}$

(A3) $$dx_{t}^{i}=a^{i}\left(x_{t},t\right)dt+b^{ik}\left(x_{t},t\right)\circ dw_{t}^{k},\;\;\;\;\;A^{i}\left(x_{t},t\right)=a^{i}\left(x_{t},t\right)+\frac{1}{2}b^{jk}\left(x_{t},t\right)\nabla_{j}b^{ik}\left(x_{t},t\right)$$

Here, and further in this appendix, $\nabla_{j}$ denotes the partial derivative $\partial /\partial x^{j}.$ The relation between Ito drift $A^{i}$ and Stratanovich drift $a^{i}$ can be easily obtained by expanding $b^{ik}$ into a series and substituting γ=1/2 and $dw_{t}^{l}dw_{t}^{k}=\delta^{lk}dt$. The Stratanovich SDE for the time-reversed process (A2) is given by a similar equation

(A4) $$d{\bar{x}}_{\bar{t}}^{i}={\bar{a}}^{i}\left({\bar{x}}_{\bar{t}},\bar{t}\right)d\bar{t}+{\bar{b}}^{ik}\left({\bar{x}}_{\bar{t}},\bar{t}\right)\circ d\;{\bar{w}}_{\bar{t}}^{k},\;\;\;\;{\bar{A}}^{i}\left({\bar{x}}_{\bar{t}},\bar{t}\right)={\bar{a}}^{i}\left({\bar{x}}_{\bar{t}},\bar{t}\right)+\frac{1}{2}{\bar{b}}^{jk}\left({\bar{x}}_{\bar{t}},\bar{t}\right)\nabla_{j}{\bar{b}}^{ik}\left({\bar{x}}_{\bar{t}},\bar{t}\right)$$

where $d{\bar{w}}_{\bar{t}}^{l}d{\bar{w}}_{\bar{t}}^{k}=\delta^{lk}d\bar{t}$ and $\bar{\gamma }=1-\gamma =1/2$ are used. Note that these are the Stratanovich drift coefficients ${\bar{a}}^{i}$ and $a^{i}$ that are treated here as physical quantities, while the Ito drift coefficients ${\bar{A}}^{i}$ and $A^{i}$ involve mathematical corrections merely reflecting temporal asymmetry of the Ito formulation. As one can see below, the physical drift term and the diffusion-induced drift adjustment change differently under time reversal.

It is also worthwhile to consider the process ${\bar{x}}_{\bar{t}}$ defined by (A4) as being parametrised by direct time $t=-\bar{t}$, that is ${\bar{x}}_{t}\left(t\right)={\bar{x}}_{\bar{t}}\left(-t\right)$. Note that ${\bar{x}}_{t}$ is generally different from $x_{t}$. In line with antisymmetric time reversal (9), we can assume

(A5) $${\bar{a}}^{i}\left(x,-t\right)=-a^{i}\left(x,t\right),\;\;\;\;\;{\bar{b}}^{ij}\left(x,-t\right)=-b^{ij}\left(x,t\right)$$

use $dt=-d\bar{t}$ and $\bar{d}w_{t}^{k}=-\bar{d}w_{\bar{t}}^{k}$ to obtain

(A6) $$d{\bar{x}}_{t}^{i}=a^{i}\left({\bar{x}}_{t},t\right)dt+b^{ik}\left({\bar{x}}_{t},t\right)\circ d{\bar{w}}_{t}^{k}$$

This is still reverse-time process ${\bar{x}}_{t}$, but parametrised by *t* instead of $\bar{t},$ while the processes $x_{t}$ and ${\bar{x}}_{t}$ may or may not be equivalent. The apparent similarity of Equations (A6) and (A3) is deceiving: note that $dw_{t}^{l}dw_{t}^{k}=\delta^{lk}dt$ in (A3) but $d{\bar{w}}_{t}^{l}d{\bar{w}}_{t}^{k}=\left(-d{\bar{w}}_{\bar{t}}^{l}\right)\left(-d{\bar{w}}_{\bar{t}}^{k}\right)=\delta^{lk}d\bar{t}=-\delta^{lk}dt.$ This is a principal change associated with time reversal of diffusion, which cannot be removed by a mere replacement of variables.

Note that the antisymmetric assumption ${\bar{a}}^{i}=-a^{i}$ is consistently applied here to the Stratanovich drifts while the Ito drifts ${\bar{A}}^{i}\neq -A^{i}$ are generally different, assuming ${\bar{A}}^{i}=-A^{i}$ would constitute a different physical assumption that would result in a more restrictive understanding and equations.

### Appendix A.2. Transitional Probabilities and Markov Properties

The *Markov property* is fundamental to the stochastic equations and is conventionally interpreted in time-directional manner: the future does not depend on the past when the present is given. Note that this also means that the past is independent of the future when the present is given. Assuming $t_{1}<t<t_{2}$ we can write for conditional probabilities of $x_{t}$

(A7) $$P\left(x_{2},t_{2}|x_{1},t_{1};x,t\right)=P\left(x_{2},t_{2}|x,t\right)$$

or equivalently

(A8) $$P\left(x_{1},t_{1}|x,t;x_{2},t_{2}\right)=P\left(x_{1},t_{1}|x,t\right).$$

Here, ($x_{k},t_{k}$) denotes condition $x_{t}\left(t_{k}\right)=x_{k}$ and $P\left(A|B\right)$ represents the conditional (or transitional in the Markov context) probability of event A given event B. Note that independence of the future from the past also means that the past does not depend on the future when the present is given. Hence, both Equations (A7) and (A8) are valid for both processes. In time-symmetric interpretations, the Markov property resembles properties of Gibbs fields [26] on linear topology: this property implies that for any $t_{0}<t_{1}<t<t_{2}<t_{3}$ by

(A9) $$P\left(x,t|x_{0},t_{0};x_{1},t_{1};x_{2},t_{2};x_{3},t_{3}\right)=P\left(x,t|x_{1},t_{1};x_{2},t_{2}\right)$$

While the Markov property is objectively time-symmetric, all principal formulations of stochastic calculus—the Ito, Stratonovich, and Skorokhod versions—incorporate at least some time-directionality, albeit to different extents.

The application of conditional version of the probabilistic rules to the process $x_{t}$ within, assuming that the times are ordered as $t_{1}<t<t_{2}$, gives

(A10) $$P\left(x,t|x_{1},t_{1};x_{2},t_{2}\right)=P\left(x_{2},t_{2}|x_{1},t_{1};x,t\right)\frac{P\left(x,t|x_{1},t_{1}\right)}{P\left(x_{2},t_{2}|x_{1},t_{1}\right)}=\frac{P\left(x_{2},t_{2}|x,t\right)P\left(x,t|x_{1},t_{1}\right)}{P\left(x_{2},t_{2}|x_{1},t_{1}\right)}$$

which uses Markov property (A7) and reflects conventional probability manipulations according to Bayes theorem $P\left(A|B\right)=P\left(B|A\right)P\left(A\right)/P\left(B\right)$. We also need a similar expression for the reverse-time process ${\bar{x}}_{t}$: note that the Markov properties (A7)–(A9) are equivalently valid for ${\bar{x}}_{t}.$ Equation (A10) then becomes

(A11) $$P\left(\bar{x},t|{\bar{x}}_{1},t_{1};{\bar{x}}_{2},t_{2}\right)=\frac{P\left({\bar{x}}_{1},t_{1}|\bar{x},t\right)P\left(\bar{x},t|{\bar{x}}_{2},t_{2}\right)}{P\left({\bar{x}}_{1},t_{1}|{\bar{x}}_{2},t_{2}\right)}$$

where Markov property (A8) is applied to the process ${\bar{x}}_{t}$ and (${\bar{x}}_{k},t_{k}$) denotes the condition ${\bar{x}}_{t}\left(t_{k}\right)=x_{k}$.

### Appendix A.3. Kolmogorov Equations for Transitional Probabilities

The transitional probabilities for a Markov diffusion process depend only on diffusion and drift coefficients and on the conditions that explicitly define these probabilities but not on any other initial or final conditions that may constrain the overall distribution. The transitional probabilities $\varphi \left(x,t;x_{1},t_{1}\right)=P\left(x,t|x_{1},t_{1}\right)$ and $\psi \left(x,t;x_{2},t_{2}\right)=P\left(x_{2},t_{2}|x,t\right)$ of the direct-time Markov diffusion process $x_{t}$ defined by Ito SDE (A1), respectively, satisfy the following *Kolmogorov forward and backward equations* [17]

(A12) $$\frac{\partial \varphi }{\partial t}=-\nabla_{i}\left(A^{i}\varphi \right)+\nabla_{i}\nabla_{j}\left(B^{ij}\varphi \right),\;\;\mathrm{with}\;\;{\left(\varphi \right)}_{t=t_{1}}=\delta \left(x-x_{1}\right)$$

(A13) $$-\frac{\partial \psi }{\partial t}=A^{i}\nabla_{i}\psi +B^{ij}\nabla_{i}\nabla_{j}\psi ,\;\;\mathrm{with}\;\;{\left(\psi \right)}_{t=t_{2}}=\delta \left(x-x_{2}\right)$$

where $B^{ij}=b^{ik}b^{jk}/2$ is symmetric. Here and throughout the paper, we imply $\varphi =\varphi \left(x,t\right)$ and $\psi =\psi \left(x,t\right),$ meaning that derivatives are applied to the first two arguments of the functions. The functional arguments may be omitted when the context makes this clear. The forward (A12) and backward (A13) equations are formulated for the direct-time Markov diffusion process $x_{t}$ (A1), have adjoint operators on their right-hand sides, and should not be confused with the corresponding equations for the reverse-time Markov diffusion process ${\bar{x}}_{t}\left(t\right)={\bar{x}}_{\bar{t}}\left(-t\right)$. The reverse-time Markov diffusion process ${\bar{x}}_{t}$ defined by Ito SDE (A2) has Kolmogorov forward and backward equations for corresponding transitional probabilities $\bar{\varphi }=\bar{\varphi }\left(x,t\right)=\bar{\varphi }\left(x,t;x_{2},t_{2}\right)=P\left(\bar{x},t|{\bar{x}}_{2},t_{2}\right)$ and $\bar{\psi }=\bar{\psi }\left(x,t\right)=\bar{\psi }\left(x,t;x_{1},t_{1}\right)=P\left({\bar{x}}_{1},t_{1}|\bar{x},t\right)$ given by

(A14) $$-\frac{\partial \bar{\varphi }}{\partial t}=-\nabla_{i}\left({\bar{A}}^{i}\bar{\varphi }\right)+\nabla_{i}\nabla_{j}\left({\bar{B}}^{ij}\bar{\varphi }\right),\;\;\mathrm{with}\;\;{\left(\bar{\varphi }\right)}_{t=t_{2}}=\delta \left(x-x_{2}\right)$$

(A15) $$\frac{\partial \bar{\psi }}{\partial t}={\bar{A}}^{i}\nabla_{i}\bar{\psi }+{\bar{B}}^{ij}\nabla_{i}\nabla_{j}\bar{\psi },\;\;\mathrm{with}\;\;{\left(\bar{\psi }\right)}_{t=t_{1}}=\delta \left(x-x_{1}\right)$$

where ${\bar{B}}^{ij}={\bar{b}}^{ik}{\bar{b}}^{jk}/2$. These equations are generally different from those for the direct-time process $x_{t}.$

In the Stratanovich interpretation, the Kolmogorov equations are transformed into

(A16) $$\frac{\partial \varphi }{\partial t}=-\nabla_{i}\left(a^{i}\varphi \right)+\nabla_{i}\left(\frac{b^{ik}}{2}\nabla_{j}\left(b^{jk}\varphi \right)\right),\;\;\;\;-\frac{\partial \psi }{\partial t}=a^{i}\nabla_{i}\psi +b^{ik}\nabla_{i}\left(\frac{b^{jk}}{2}\nabla_{j}\psi \right)$$

for the direct-time process $x_{t}$ and into

(A17) $$-\frac{\partial \bar{\varphi }}{\partial t}=-\nabla_{i}\left({\bar{a}}^{i}\bar{\varphi }\right)+\nabla_{i}\left(\frac{{\bar{b}}^{ik}}{2}\nabla_{j}\left({\bar{b}}^{jk}\bar{\varphi }\right)\right),\;\;\;\;\frac{\partial \bar{\psi }}{\partial t}={\bar{a}}^{i}\nabla_{i}\bar{\psi }+{\bar{b}}^{ik}\nabla_{i}\left(\frac{{\bar{b}}^{jk}}{2}\nabla_{j}\bar{\psi }\right)$$

for the reverse-time process ${\bar{x}}_{t},$ where we substitute the definitions of $A^{i},$ $B^{ij},$ ${\bar{A}}^{i}$ and $\;{\bar{B}}^{ij}$. The boundary conditions imposed on φ, ψ, $\bar{\varphi }$ and $\bar{\psi }$ remain the same as in Equations (A12)–(A15).

Note that according to the definitions of the transitional probabilities φ, ψ, $\bar{\varphi }$ and $\bar{\psi }$ require that for any $t_{1}<t_{2}$

(A18) $$\varphi \left(x_{2},t_{2};x_{1},t_{1}\right)=\psi \left(x_{1},t_{1};x_{2},t_{2}\right),\;\;\;\bar{\varphi }\left(x_{1},t_{1};x_{2},t_{2}\right)=\bar{\psi }\left(x_{2},t_{2};x_{1},t_{1}\right)$$

While Kolmogorov forward and backward equations specifically govern transitional probabilities of a Markov diffusion process, the Fokker–Planck equation governs diffusive evolution of a more general class of probabilistic distributions. The forms of the Fokker–Planck and Kolmogorov forward equations coincide and these terms are often used synonymously, but there still remains a subtle difference between them. While φ and ψ, $\bar{\varphi }$ and $\bar{\psi }$ represent transitional probabilities, physical interpretation of equations involving forward and backward parabolicities may differ in different applications. For example, in Conditional Moment Closure [27], (A14) corresponds to a probability density function, while (A15) governs the conditional expectation of a scalar.

### Appendix A.4. Markov Diffusion Bridge

At this stage, we turn from consideration of Markov diffusion families, semigroups, and transitional probabilities to specific processes that are subject to fixed initial and/or final conditions. While realisations of Wiener process are time-symmetric—Wiener process viewed in reverse time is still an equivalent Wiener process– processes $x_{t}$ and ${\bar{x}}_{t}$ may or may not have similar time-symmetric statistics even if the temporal boundary conditions imposed on them are time-symmetric. The term *bridge* is conventionally used to emphasise setting two conditions—initial and final—instead of the common preference for initial conditions only, which is usually justified by antecedent causality. We impose the same temporal boundary conditions on both $x_{t}$ and ${\bar{x}}_{t}$

(A19) $$\begin{array}{cc} {\left({\bar{x}}_{t}\right)}_{t=t_{1}} & ={\left(x_{t}\right)}_{t=t_{1}}=x_{1} \end{array}$$

(A20) $$\begin{array}{cc} {\left({\bar{x}}_{t}\right)}_{t=t_{2}} & ={\left(x_{t}\right)}_{t=t_{2}}=x_{2} \end{array}$$

From now on, $x_{1},t_{1},x_{2}t$ and $t_{2}$ are fixed parameters. At this point, we define a new function *f* that corresponds to the bridge-conditioned probability $f\left(x,t;x_{1},t_{1};x_{2},t_{2}\right)=P\left(x,t|x_{1},t_{1};x_{2},t_{2}\right),$ so that Equation (A10) takes the form

(A21) $$f\left(x,t;x_{1},t_{1};x_{2},t_{2}\right)=\frac{\psi \left(x,t;x_{2},t_{2}\right)\varphi \left(x,t;x_{1},t_{1}\right)}{C\left(x_{2},t_{2};x_{1},t_{1}\right)}$$

where *C* is defined by $C\left(x_{2},t_{2};x_{1},t_{1}\right)=P\left(x_{2},t_{2}|x_{1},t_{1}\right),$ that is $C=\varphi \left(x_{2},t_{2};x_{1},t_{1}\right)=\psi \left(x_{1},t_{1};x_{2},t_{2}\right)$. For the distribution of the reverse process $\bar{f}\left(x,t;x_{1},t_{1};x_{2},t_{2}\right)=$ $P\left(\bar{x},t|{\bar{x}}_{1},t_{1};{\bar{x}}_{2},t_{2}\right),$ Equation (A11) takes the form

(A22) $$\bar{f}\left(x,t;x_{1},t_{1};x_{2},t_{2}\right)=\frac{\bar{\psi }\left(x,t;x_{1},t_{1}\right)\bar{\varphi }\left(x,t;x_{2},t_{2}\right)}{\bar{C}\left(x_{1},t_{1};x_{2},t_{2}\right)}$$

where $\bar{C}$ is defined by $\bar{C}\left(x_{1},t_{1};x_{2},t_{2}\right)=P\left({\bar{x}}_{1},t_{1}|{\bar{x}}_{2},t_{2}\right)$ (implying $\bar{C}=\bar{\varphi }\left(x_{1},t_{1};x_{2},t_{2}\right)=\bar{\psi }\left(x_{2},t_{2};x_{1},t_{1}\right)$).

The model specified by (A3) or (A6) in conjunction with (A19) and (A20) can also be referred to as a *Schrödinger bridge* [28], which usually refers to a broader range of problems associated with finding probability density ρ(x,t) that is compliant with the initial and terminal conditions

(A23) $$\rho {\left(x,t\right)}_{t=t_{1}}=\rho_{1}\left(x\right)\;\mathrm{and}\;\rho {\left(x,t\right)}_{t=t_{2}}=\rho_{2}\left(x\right)$$

The modern interpretation of Schrödinger bridges has evolved towards broader understanding of the problem including transport optimisation. Given conditional probability $f\left(x,t;x_{1},t_{1};x_{2},t_{2}\right)$ and Markov property (A9), the solution of (A23) can be evaluated by convolution of *f* with $\rho_{1}\left(x\right)$ and $\rho_{2}\left(x\right)$, as demonstrated by Schrödinger in their principal work [28]. While discussion of a broader spectrum of issues associated with Schrödinger bridges is outside the scope of the present publication, it can be found in the excellent overview by Chetrite et al. [29].

### Appendix A.5. Doob’s Transform and Anderson’s Reversal for the Markov Bridge

In this section, we assume that the processes x(t) and $\bar{x}\left(t\right)$ have the same drift and diffusion coefficients as specified by antisymmetric relations in (A5) in conjunction with the Stratanovich interpretation $\gamma =\bar{\gamma }=1/2$ of the SDEs. The question becomes whether this ensures that the processes $x_{t}$ and ${\bar{x}}_{t},$ which are subject to the same boundary conditions (A19) and (A20), are equivalent, and, therefore, have the same distributions *f* and $\bar{f}$. The answer is generally negative. Indeed, let us assume that $f=\bar{f}$ is a smooth solution in the domain $t_{1}\leq t\leq t_{2}$ and create a small variation δa of drift a mostly concentrated in a small vicinity of a selected point $x_{0},t_{0},$ within the domain (i.e., $t_{1}<t_{0}<t_{2}$) so that $\left|\nabla \cdot \delta a\right|\gg \left|\delta a\right|$. This alteration would affect functions φ and $\bar{\varphi }$ much more than ψ and $\bar{\psi }$, since the former but not the latter are governed by conservative equations. These local disturbances $\delta \varphi =\delta \bar{\varphi }$ are created by the same drift variation preserving the similarity of the overall distributions $\delta f=\delta \bar{f}$. These disturbances, however, would diffuse downstream $t>t_{0}$ from $x_{0},t_{0}$ for δφ and upstream $t<t_{0}$ from $x_{0},t_{0}$ for $\delta \bar{\varphi }$ violating the similarity of *f* and $\bar{f}$. If f≠ $\bar{f}$, then the direct-time and reverse-time processes are generally different and require selection of a preferred time direction to describe the laws of nature. Having a preferred direction of time corresponds to some form of antecedent causality.

According to (A12) and (A13), $f=f\left(x,t\right)$ satisfies the following equation

(A24) $$\frac{\partial f}{\partial t}+\nabla_{i}\left(a^{i}f\right)=\nabla_{i}\left(\frac{b^{ik}\psi }{2C}\nabla_{j}\left(b^{jk}\varphi \right)-\frac{b^{ik}b^{jk}\varphi }{2C}\nabla_{j}\psi \right)=\nabla_{i}\left(\frac{b^{ik}}{2}\nabla_{j}\left(b^{jk}f\right)-\alpha_{D}^{i}f\right)$$

where

(A25) $$a_{D}^{i}=a^{i}+\alpha_{D}^{i},\;\;\;\alpha_{D}^{i}=\frac{b^{ik}b^{jk}}{\psi }\nabla_{j}\psi =b^{ik}b^{jk}\nabla_{j}\ln\psi =2B^{ij}\nabla_{j}\ln\psi$$

and

(A26) $$\left(a\right)\;{\left(f\right)}_{t=t_{1}}=\delta \left(x-x_{1}\right)\;\;\;\mathrm{while}\;\left(b\right)\;\;{\left(f\right)}_{t=t_{2}}={\left(\psi \right)}_{t=t_{2}}=\delta \left(x-x_{2}\right)$$

Equation (A24) is interpreted as a direct-time *Kolmogorov forward (Fokker–Planck) equation* with *Stratanovich* diffusion $b^{jk}$ and drift $a_{D}^{i}=a^{i}+\alpha_{D}^{i}$ coefficients, where *f* is the transitional probability from the state $x_{t}\left(t_{1}\right)=x_{1}$ to the state $x_{t}\left(t\right).$ While imposing the initial condition (A26) (a) on *f* remains essential, the final condition (A26) (b) is automatically satisfied due to the additional drift component $\alpha_{D}^{i},$ which represents *Doob’s correction drift*. Note that, due to absence of time reversal, this correction is the same for both Stratanovich and Ito formulations. Equation (A24) implements *Doob’s h-transform* [30] in direct Kolmogorov equation, where the conditioning function h=ψ is used to enforce the final conditions (A26) (b) upon *f*.

Now, we turn to consider the reverse-time process ${\bar{x}}_{t}$ and its distribution function $\bar{f}=\bar{f}\left(x,t\right)$, which, according to (A14) and (A15), satisfies the following equation

(A27) $$\frac{\partial \bar{f}}{\partial t}+\nabla_{i}\left(a^{i}\bar{f}\right)=\nabla_{i}\left(\frac{b^{ik}b^{jk}\bar{\varphi }}{2\bar{C}}\nabla_{j}\bar{\psi }-\frac{\bar{\psi }b^{ik}}{2\bar{C}}\nabla_{j}\left(b^{jk}\bar{\varphi }\right)\right)=\nabla_{i}\left(\frac{b^{ik}}{2}\nabla_{j}\left(b^{jk}\bar{f}\right)-\alpha_{A}^{i}\bar{f}\right)$$

where

(A28) $$a_{A}^{i}=a^{i}+\alpha_{A}^{i}=-{\bar{a}}^{i}+\alpha_{A}^{i},\;\;\;\alpha_{A}^{i}=\frac{b^{ik}}{\bar{\varphi }}\nabla_{j}\left(b^{jk}\bar{\varphi }\right)=b^{ik}\nabla_{j}b^{jk}+b^{ik}b^{jk}\nabla_{j}\ln\bar{\varphi }$$

and

(A29) $$\left(a\right)\;{\left(\bar{f}\right)}_{t=t_{1}}=\delta \left(x-x_{1}\right)\;\;\;\mathrm{while}\;\;\;\left(b\right)\;{\left(\bar{f}\right)}_{t=t_{2}}={\left(\bar{\varphi }\right)}_{t=t_{2}}=\delta \left(x-x_{2}\right)$$

For the sake of comparison with (A24), Equation (A27) characterising the reverse-time process is written as direct-time Fokker–Planck with Stratanovich diffusion $b^{ik}$ and drift $a_{A}^{i}=a^{i}+\alpha_{A}^{i}$ coefficients and, therefore, involves a time reversal. The additional drift $\alpha_{A}^{i}$ specified by (A28) is *Stratanovich* form of the *Anderson’s correction drift*. This correction ensures a consistent antisymmetric time reversal of a Markov diffusion process [18], although here we reverse the reverse-time process ${\bar{x}}_{t}$ to run it forward in time—let us denote this process ${\bar{\bar{x}}}_{t}.$ We emphasise that this reversal is not only a reversal of the Fokker–Planck equation for $\bar{f}$ that is consistent with the behaviour of $\bar{\varphi }$ at the final state, but also time reversal of the underlying stochastic process. According to the *Anderson theorem* [18], which goes beyond an earlier result by Stratanovich [31], processes ${\bar{x}}_{t}$ and ${\bar{\bar{x}}}_{t}$ are equivalent—this theorem guaranties not only probabilistic equivalence of the processes obtained by Anderson’s reversal but, under specified conditions, also does this in a strong sense implying equivalence of the original and time-reversed stochastic trajectories.

Equation (A27) governs probability distribution $\bar{f}$ for both processes ${\bar{x}}_{t}$ and ${\bar{\bar{x}}}_{t}$, so that $\bar{f}$ automatically satisfies the final condition (A29) (b). Due to the initial condition (A29) (a), this $\bar{f}$ can be interpreted as the transitional probability from the state ${\bar{\bar{x}}}_{t}\left(t_{1}\right)=x_{1}$ to the state ${\bar{\bar{x}}}_{t}\left(t\right)$ and (A27) as the corresponding direct-time *Kolmogorov forward equation*. While processes ${\bar{x}}_{t}$ and ${\bar{\bar{x}}}_{t}$ are equivalent, this is not necessarily the case for ${\bar{x}}_{t}$ and $x_{t}$—these processes have not been converted using Anderson’s reversal and, indeed, Equations (A24) and (A27) can have different drifts $a_{A}^{i}\neq a_{D}^{i}$ when $\alpha_{D}^{i}\neq \alpha_{A}^{i}$.

The expressions for Anderson’s drift $\alpha_{A}^{i}$ and Doob’s drift $\alpha_{D}^{i}$ are similar but not the same—these two approaches enforce the same final conditions but do this differently, with and without the reversal of time. If, however, $\alpha_{D}^{i}=\alpha_{A}^{i},$ then the Kolmogorov forward Equations (A24) and (A27) have exactly the same diffusion $b^{ik}$ and drift $a^{i}+\alpha_{D}^{i}$ coefficients and the same initial conditions, then the underlying stochastic processes $x_{t}$ and ${\bar{x}}_{t}$ are also equivalent. Therefore, $f=\bar{f}$ when $\alpha_{D}^{i}=\alpha_{A}^{i}$ so that:

(A30) $$\frac{b^{ik}b^{jk}}{\psi }\nabla_{j}\psi =\frac{b^{ik}}{\bar{\varphi }}\nabla_{j}\left(b^{jk}\bar{\varphi }\right)$$

While Equations (A24) and (A27) are formulated to evolve forward in time, we could have selected the reverse time $\bar{t}$ for comparison of *f* and $\bar{f}$—our formulation is fully time-symmetric. This would lead to the complimentary condition

(A31) $$\frac{b^{ik}b^{jk}}{\bar{\psi }}\nabla_{j}\bar{\psi }=\frac{b^{ik}}{\varphi }\nabla_{j}\left(b^{jk}\varphi \right)$$

which can also be derived from $f=\bar{f}$ and (A30).

Our analysis results in the following theorem:

**Theorem** **A1.**
*With consistent choice of the diffusion and drift coefficients, the direct-time and reverse-time formulations of the Markov diffusion bridge problem are fully time-symmetric and equivalent if and only if Doob’s transform and Anderson’s reversal correction drifts coincide.*

Indeed, Anderson’s theorem establishes the fundamental equivalence of the reverse-time process ${\bar{x}}_{\bar{t}}$ and its time reversal ${\bar{\bar{x}}}_{t}$ running forward in time—both processes have the same distribution $\bar{f}\left(x,t\right).$ When Anderson’s and Doob’s drifts coincide $\alpha_{D}^{i}=\alpha_{A}^{i}$, the processes ${\bar{\bar{x}}}_{t}$ and $x_{t}$ have the same initial condition, and the same drift $a_{D}^{i}=a_{A}^{i}$ and diffusion $b^{ij}$ coefficients in the direct-time Kolmogorov forward equations and, therefore, must be equivalent. If $\alpha_{D}^{i}\neq \alpha_{A}^{i},$ then the processes have different drift coefficients and, therefore, are not equivalent irrespective whether *f* and $\bar{f}$ coincide or not. This proves the theorem.

The consistency of drift and diffusion coefficients in direct-time and reverse-time models specified by (A5) appears to be insufficient to ensure that Equations (A24) and (A27) are exactly the same due to possible differences between Doob’s and Anderson’s correction drifts, $\alpha_{D}^{i}$ and $\alpha_{A}^{i}$. The equivalence of Equations (A24) and (A27) and the corresponding processes can nevertheless be achieved but requires additional conditions that are discussed in the next subsection.

### Appendix A.6. Antisymmetric Time Reversal and Odd Symmetry

In line with antisymmetric time reversal (9), (A5), and due to physical reasons discussed in the main text, we restrict our attention to the case

(A32) $${\bar{a}}^{i}=-a^{i},\;\;{\bar{b}}^{ij}=-b^{ij},\;\;\nabla_{i}b^{ik}=0,\;\;\nabla_{i}a^{i}=0\;\;$$

which ensure that

(A33) $$\phi \left(x,t;x_{1},t_{1}\right)=\bar{\psi }\left(x,t;x_{1},t_{1}\right),\;\;\;\psi \left(x,t;x_{2},t_{2}\right)=\bar{\phi }\left(x,t;x_{2},t_{2}\right)$$

(A34) $$\bar{f}\left(x,t;x_{1},t_{1};x_{2},t_{2}\right)=f\left(x,t;x_{1},t_{1};x_{2},t_{2}\right),\;\;\bar{C}=C$$

These conditions are important for entropy-consistent formulation of the model that preserves the phase volume and *odd symmetry* implies in this work that these conditions are satisfied. With conditions (A32), Equations (A16) and (A17) are transformed to take the form of the Markov bridge model with odd symmetry:

(A35) $$\frac{\partial \varphi }{\partial t}=L\varphi ,\;\;\;-\frac{\partial \psi }{\partial t}=\bar{L}\psi ,\;\;\;-\frac{\partial \bar{\varphi }}{\partial t}=\bar{L}\bar{\varphi },\;\;\;\frac{\partial \bar{\psi }}{\partial t}=L\bar{\psi }$$

where new adjoint operators

(A36) $$L\left[\cdot \right]=-\nabla_{i}\left(a^{i}\left[\cdot \right]\right)+\nabla_{i}\left(B^{ij}\nabla_{j}\left[\cdot \right]\right)\;\;\mathrm{and}\;\;\bar{L}\left[\cdot \right]=+\nabla_{i}\left(a^{i}\left[\cdot \right]\right)+\nabla_{i}\left(B^{ij}\nabla_{j}\left[\cdot \right]\right)\;\;$$

are introduced. Under these conditions, the dynamic the direct-time and reverse-time model are fully equivalent. Due to (A18) and (A33), this model is complaint with the odd-symmetric analogue

(A37) $$\bar{\varphi }\left(x_{1},t_{1};x_{2},t_{2}\right)=\varphi \left(x_{2},t_{2};x_{1},t_{1}\right),\;\;\;\bar{\psi }\left(x_{2},t_{2};x_{1},t_{1}\right)=\psi \left(x_{1},t_{1};x_{2},t_{2}\right)$$

of the even-symmetric detailed balance considered in Appendix B. Note that Equations (A35) and (A36) allow only for constant steady-state solutions (see Section 3.1).

The key point is presented in the form of the proposition:

**Proposition** **A1.**
*Doob’s transform and Anderson’s reversal corrections coincide under odd-symmetric conditions specified in (A32). Therefore, under these conditions, the Markov bridge problem is fully time-symmetric and the direct-time and reverse-time formulations of the problem are equivalent.*

Indeed, substitution of the odd-symmetric conditions of (A32) into Equations (A16) and (A17) with boundary conditions taken from (A12)–(A15) results in $\bar{\varphi }=\psi$ and $\bar{\psi }=\varphi ,$ which, with the use of $\nabla_{i}b^{ik}=0,$ ensures that condition (A30) is satisfied. Therefore, $\alpha_{D}^{i}=\alpha_{A}^{i}$ in (A24) and (A27) and, according to Theorem A1, the problem is time-symmetric, implying that solving the Markov bridge problem for $x_{t}$ or for ${\bar{x}}_{\bar{t}}$ is mathematically and physically equivalent.

Note that condition (A30) represents *n* scalar equations i=1,...,n (where *n* is the number of variables) with $n^{2}$ independent input values in $b^{ik}$ with only two scalar functions ψ and $\bar{\varphi }$ to satisfy it—in general, Equation (A30) is overdetermined. While constrains (A32) are sufficient for proper antisymmetric reversibility, are they necessary to ensure that $\alpha_{D}^{i}=\alpha_{A}^{i}$? The answer is negative and the class of solutions satisfying $\alpha_{D}^{i}=\alpha_{A}^{i}$ is substantially wider. For example, it is easy to see that if

(A38) $$b^{ik}={\hat{b}}^{ik}\left(x,t\right)\beta \left(x\right),\;\;\;\;\nabla_{i}{\hat{b}}^{ik}=0,\;\;\nabla_{i}\frac{a^{i}}{\beta }=0$$

then the conditions (A30) and (A31) as well as Equations (A16) and (A17) are satisfied by

(A39) $$\psi =c_{2}\beta \bar{\varphi },\;\;c_{2}=\frac{1}{\beta (x_{2})},\;\;\bar{\psi }=c_{1}\beta \varphi ,\;\;\;c_{1}=\frac{1}{\beta (x_{1})}=c_{2}\frac{\bar{C}}{C}$$

## Appendix B. Kolmogorov’s Concept of Symmetric Reversibility

Kolmogorov [17] considered a *Markov diffusion process* $x_{t}$ with a steady-state distribution $\eta \left(x\right)$ satisfying the Fokker–Planck equation in (A12)

(A40) $$0=\frac{\partial \eta }{\partial t}=-\nabla_{i}\left(A^{i}\left(x\right)\eta \right)+\nabla_{i}\nabla_{j}\left(B^{ij}\left(x\right)\eta \right)$$

under conditions when the drift and diffusion coefficients $A^{i}\left(x\right)$ and $B^{ij}\left(x\right)$ do not depend on time.

### Appendix B.1. Symmetric Time Reversal

The symmetric time reversal of the process $x_{t}$ produces a new process ${\bar{x}}_{\bar{t}}$ that that depends on $\bar{t}=-t$ in the same way that $x_{t}$ depends on *t*. The reverse-time process can also be parametrised by *t* instead of $\bar{t}$ and denoted by ${\bar{x}}_{t}$—this can more convenient for some purposes. The reverse-time process must also have a steady-state distribution $\bar{\eta }\left(x\right)$

(A41) $$0=\frac{\partial \bar{\eta }}{\partial \bar{t}}=-\nabla_{i}\left({\bar{A}}^{i}\left(x\right)\bar{\eta }\right)+\nabla_{i}\nabla_{j}\left({\bar{B}}^{ij}\left(x\right)\bar{\eta }\right)$$

which, due to the symmetric constrains

(A42) $${\bar{A}}^{i}\left(x\right)=A^{i}\left(x\right),\;\;{\bar{B}}^{ij}\left(x\right)=B^{ij}\left(x\right),\;\;\bar{\eta }\left(x\right)=\eta \left(x\right)$$

coincides with the steady-state distribution of the direct-time process $\eta \left(x\right)$.

The similarity between the direct-time and reverse-time processes implies the following conditions

(A43) $$\varphi \left(x,t;x_{1},t_{1}\right)=\bar{\psi }\left(x_{1},t;x,t_{1}\right);\;\;\;\;\bar{\varphi }\left(x,t;x_{2},t_{2}\right)=\psi \left(x_{2},t;x,t_{2}\right)$$

Since the drift and diffusion coefficients are time-independent, the functions φ, $\bar{\psi }$, $\bar{\varphi }$, and ψ depend only on time differences, i.e., on $t-t_{1}$ or $t_{2}-t$, assuming $t_{1}\leq t\leq t_{2}$ but, in order to avoid confusion, it is more convenient to retain the original representation of these functions. Indeed, by definition in Section A.3, $\varphi \left(x,t;x_{1},t_{1}\right)=P\left(x,t|x_{1},t_{1}\right)$ is the transition probability from $x_{t}=x_{1}$ to $x_{t}=x$ over time interval $t-t_{1}$, while $\bar{\psi }\left(x_{1},t;x,t_{1}\right)=P\left(\bar{x},t_{1}|{\bar{x}}_{1},t\right)$ is the transition probability from ${\bar{x}}_{t}=x_{1}$ to ${\bar{x}}_{t}=x$ over the same time interval $t-t_{1}$ back in time. These transitional probabilities must be the same due to equivalence of $x_{t}$ and ${\bar{x}}_{\bar{t}}$. The second equality in (A43) has the same justification: $\bar{\varphi }\left(x,t;x_{2},t_{2}\right)=P\left(\bar{x},t|{\bar{x}}_{2},t_{2}\right)$ and $\psi \left(x_{2},t;x,t_{2}\right)=P\left(x,t_{2}|x_{2},t\right)$.

### Appendix B.2. The Detailed Balance Condition

According to Kolmogorov [17], the process $x_{t}$ is called *reversible* (i.e., evenly reversible or *Kolmogorov-reversible*) when it satisfies the detailed balance conditions. Due to equivalence of $x_{t}$ and ${\bar{x}}_{\bar{t}},$ the reverse-time process must also satisfy similar conditions. With the use of new functions

(A44) $$\phi \left(x,t;x_{1},t_{1}\right)\overset{\mathrm{def}}{=}\frac{\eta \left(x_{1}\right)}{\eta \left(x\right)}\varphi \left(x,t;x_{1},t_{1}\right),\;\;\;\bar{\phi }\left(x,t;x_{2},t_{2}\right)\overset{\mathrm{def}}{=}\frac{\eta \left(x_{2}\right)}{\eta \left(x\right)}\bar{\varphi }\left(x,t;x_{2},t_{2}\right)$$

the detailed balance conditions can be expressed as

(A45) $$\left(a\right)\;\phi \left(x,t;x_{1},t_{1}\right)=\varphi \left(x_{1},t;x,t_{1}\right),\;\;\;\left(b\right)\;\bar{\phi }\left(x,t;x_{2},t_{2}\right)=\bar{\varphi }\left(x_{2},t;x,t_{2}\right)$$

Equation (A43) implies that the detailed balance also requires that

(A46) $$\phi \left(x,t;x_{1},t_{1}\right)=\bar{\psi }\left(x,t;x_{1},t_{1}\right),\;\;\;\psi \left(x,t;x_{2},t_{2}\right)=\bar{\phi }\left(x,t;x_{2},t_{2}\right)$$

the functions ϕ and ψ are, respectively, the same as $\bar{\psi }$ and $\bar{\phi }$. These conditions are similar to the corresponding conditions derived for odd symmetry in (A33). Symmetric time reversal is considered to have *even symmetry* when it complies with the Kolmogorov reversibility conditions. Without this compliance, the equalities in (A46) are not satisfied.

The detailed balance conditions are subject to the following reversibility theorem.

**Theorem** **A2.**
*(Kolmogorov) Stationary Markov diffusion processes $x_{t}$ satisfies the detailed balance condition (A45) (a) (and is, therefore, Kolmogorov-reversible) when and only when its steady-state distribution $\eta \left(x\right)$ is compliant with*

(A47)
$$A^{i}\eta =\nabla_{j}\left(B^{ij}\eta \right)$$

Note that any steady-state solution satisfying (A47) also complies with (A40) but not vice versa. The proof of the theorem is given by Kolmogorov [17]. As the processes ${\bar{x}}_{\bar{t}}$ is a symmetric image of $x_{t}$, the Kolmogorov reversibility theorem also applies to ${\bar{x}}_{\bar{t}}$ requiring ${\bar{A}}^{i}\bar{\eta }=\nabla_{j}\left({\bar{B}}^{ij}\bar{\eta }\right)$ for validity of (A45) (b).

**Proposition** **A2.**
*Anderson’s time reversal of stationary Kolmogorov-reversible process $x_{t}$ with a steady-state distribution $\eta \left(x\right)$ is a stationary reversible process ${\bar{x}}_{\bar{t}}$ with the same steady-state distribution $\bar{\eta }\left(x\right)=\eta \left(x\right).$*

Indeed, noting (A47) and applying Anderson’s time reversal formulae [18] with our present Ito notations, we obtain

(A48) $${\bar{B}}^{ij}=B^{ij},\;\;{\bar{A}}^{i}=-A^{i}+2\frac{\nabla_{j}\left(B^{ij}\eta \right)}{\eta }=-A^{i}+2A^{i}=A^{i}$$

which coincides with (A42). Under symmetrically steady-state conditions, Anderson’s time reversal, which generally pertains to the antisymmetric type of reversal of the drift terms ${\bar{A}}^{i}∼-A^{i}$, is flipped by intensive diffusion to comply with the symmetric reversal ${\bar{A}}^{i}=A^{i}$. Note that this equation is valid only for steady-state distributions, while general time-evolving processes $x_{t}$ and ${\bar{x}}_{\bar{t}}$ with unsteady distributions are not linked by Anderson’s time reversal. Also note that the Anderson theorem [18] is by itself a general statement, which is not restricted to any specific type of symmetry. While implications of the theorem are more prominent in the case of odd symmetry, Anderson’s theorem can also be used in the case of even symmetry. Yet, the solenoidal odd-symmetric conditions in (A32) and potential even-symmetric conditions in (A47) are generally incompatible.

### Appendix B.3. Equations for the Transitional Probabilities

To obtain the even-symmetric form of the equations for transitional probabilities we substitute φ expression in terms of ϕ by (A44) into (A12) and the coefficient $A^{i}$ determined by (A47) into (A13), take into account that $B^{ij}=B^{ji}$ and obtain equations for ϕ=ϕ(x,t) and ψ=ψ(x,t):

(A49) $$\eta \left(x\right)\frac{\partial \phi }{\partial t}=\nabla_{i}\left(\eta \left(x\right)B^{ij}\left(x\right)\nabla_{j}\phi \right),\;\;\mathrm{with}\;\;{\left(\phi \right)}_{t=t_{1}}=\delta \left(x-x_{1}\right)$$

(A50) $$-\eta \left(x\right)\frac{\partial \psi }{\partial t}=\nabla_{i}\left(\eta \left(x\right)B^{ij}\left(x\right)\nabla_{j}\psi \right),\;\;\mathrm{with}\;\;{\left(\psi \right)}_{t=t_{2}}=\delta \left(x-x_{2}\right)$$

With the use of reversed time $\bar{t}=-t,$ the reverse-time model should look the same as the direct-time model. The boundary condition for ϕ follows from the boundary condition for φ in (A12) at $x=x_{1}$. Since we prefer to use *t* as time variable, Equations (A14) and (A15) then become

(A51) $$-\eta \left(x\right)\frac{\partial \bar{\phi }}{\partial t}=\nabla_{i}\left(\eta \left(x\right)B^{ij}\left(x\right)\nabla_{j}\bar{\phi }\right),\;\;\mathrm{with}\;\;{\left(\bar{\phi }\right)}_{t=t_{2}}=\delta \left(x-x_{2}\right)$$

(A52) $$\eta \left(x\right)\frac{\partial \bar{\psi }}{\partial t}=\nabla_{i}\left(\eta \left(x\right)B^{ij}\left(x\right)\nabla_{j}\bar{\psi }\right),\;\;\mathrm{with}\;\;{\left(\bar{\psi }\right)}_{t=t_{1}}=\delta \left(x-x_{1}\right)$$

where $\bar{\phi }=\bar{\phi }\left(x,t\right)$ and $\bar{\psi }=\bar{\psi }\left(x,t\right).$ As expected from the Kolmogorov reversibility theorem, Equations (A49)–(A52), which are derived from (A47), are self-adjoint and consistent with (A46), which is equivalent to the detailed balance (A45).

### Appendix B.4. Markov Diffusion Bridge Under Even-Symmetric Conditions

In this section, we consider *Markov diffusion bridge* formulation with both the initial and final temporal boundary conditions (A19) and (A20) so that the overall distribution of the direct-time (A21) and reverse-time (A22) processes are expressed in terms of functions ϕ and ψ by the formulae

(A53) $$\begin{array}{c} f\left(x,t;x_{1},t_{1};x_{2},t_{2}\right)=\frac{\eta (x)}{C_{1}}\phi \left(x,t;x_{1},t_{1}\right)\psi \left(x,t;x_{2},t_{2}\right) \end{array}$$

(A54) $$\begin{array}{c} \;\bar{f}\left(x,t;x_{1},t_{1};x_{2},t_{2}\right)=\frac{\eta (x)}{C_{2}}\bar{\phi }\left(x,t;x_{2},t_{2}\right)\bar{\psi }\left(x,t;x_{1},t_{1}\right)\;\;\;\; \end{array}$$

with the normalisation constants

(A55) $$C_{1}=\eta \left(x_{1}\right)\psi \left(x_{1},t_{1};x_{2},t_{2}\right),\;\;\;C_{2}=\eta \left(x_{2}\right)\bar{\psi }\left(x_{2},t_{2};x_{1},t_{1}\right)$$

Equations (A49)–(A52) indicate that the distributions $f=f\left(x,t\right)$ and $\bar{f}=\bar{f}\left(x,t\right)$ satisfy the equations

(A56) $$\frac{\partial f}{\partial t}=\nabla_{i}\left(\frac{\eta B^{ij}}{C_{1}}\left(\psi \nabla_{j}\phi -\phi \nabla_{j}\psi \right)\right)=\nabla_{i}\left(B^{ij}f\left(\nabla_{j}\ln\phi -\nabla_{j}\ln\psi \right)\right)$$

(A57) $$\frac{\partial \bar{f}}{\partial t}=\nabla_{i}\left(\frac{\eta B^{ij}}{C_{2}}\left(\bar{\phi }\nabla_{j}\bar{\psi }-\bar{\psi }\nabla_{j}\bar{\phi }\right)\right)=\nabla_{i}\left(B^{ij}\bar{f}\left(\nabla_{j}\ln\bar{\psi }-\nabla_{j}\ln\bar{\phi }\right)\right)$$

which can be transformed into equations

(A58) $$\frac{\partial f}{\partial t}=\nabla_{i}\left(B^{ij}\nabla_{j}f\;-\underset{\alpha_{D}^{i}}{\;\underset{︸}{B^{ij}\nabla_{j}\ln\left(\eta \psi^{2}\right)}}f\right),\;\;\;\;\;\mathrm{with}\;\;{\left(f\right)}_{t=t_{1}}=\delta \left(x-x_{1}\right)$$

(A59) $$\frac{\partial \bar{f}}{\partial t}=\nabla_{i}\left(B^{ij}\nabla_{j}\bar{f}\;-\underset{\alpha_{A}^{i}}{\;\underset{︸}{B^{ij}\nabla_{j}\ln\left(\eta {\bar{\phi }}^{2}\right)}}\bar{f}\right),\;\;\;\;\;\mathrm{with}\;\;{\left(\bar{f}\right)}_{t=t_{1}}=\delta \left(x-x_{1}\right)$$

which, as in Appendix A.5, are interpreted as direct-time Kolmogorov forward equations, where the Doob’s $\alpha_{D}^{i}$ and Anderson’s $\alpha_{A}^{i}$ corrections are the same $\alpha_{D}^{i}=\alpha_{A}^{i}$ due to (A46). Hence, Theorem A1 ensures the following proposition.

**Proposition** **A3.**
*Doob’s transform and Anderson’s reversal corrections coincide under even-symmetric conditions, which imply Kolmogorov reversibility. Therefore, the Markov bridge problem is fully time-symmetric under these conditions, making the direct-time and reverse-time formulations of the problem equivalent.*

Note that if the processes are not Kolmogorov-reversible, the direct-time $x_{t}$ and reverse-time ${\bar{x}}_{\bar{t}}$ formulations of the Markov bridge problem are generally not equivalent, even if the equations in (A42) are satisfied.
